# A real-world feasibility study: at home longitudinal use of the Cumulus NeuLogiq® platform for electrophysiological and neurocognitive measures in patients with mild Alzheimer's Disease dementia

**DOI:** 10.3389/fdgth.2026.1840966

**Published:** 2026-06-26

**Authors:** Shannon Diggin, Laura Rueda-Delgado, Alison Buick, John F. Dyer, Azar Alexander-Sefre, Florentine Barbey, James B. Rowe, Kinan Muhammed, Brian T. Harel, Bryan J. Hansen, Elizabeth Tunbridge, Robert Lai, David J. Hawellek, Ann Marie Hake, Hugh Marston, Ashwini Oswal, Elizabeth Coulthard, Vivek Pattan, Victoria Evans, Stuart Gibson, Christopher Kipps, Rouba Kozak, Nick Mannering, Mark Moss, Viet Nguyen, Kenneth Ruddock, Leslie A. Shinobu, Sofia Toniolo, Brian Murphy

**Affiliations:** 1 Cumulus Neuroscience Ltd., Belfast, United Kingdom; 2 Cumulus Neuroscience Ltd., Dublin, Ireland; 3 Department of Clinical Neurosciences, University of Cambridge, Cambridge, United Kingdom; 4 Nuffield Department of Clinical Neurosciences, University of Oxford, Oxford, United Kingdom; 5 Neuroscience Translational Medicine, Takeda Pharmaceuticals, Cambridge, MA, United States; 6 Janssen Research & Development, LLC, a Johnson & Johnson Company, Pennsylvania, PA, United States; 7 CNS Diseases Research, Boehringer Ingelheim, Biberach, Germany; 8 GSK, Stevenage, United Kingdom; 9 Pharma Research and Early Development (pRED), Hoffmann–La Roche Ltd., Basel, Switzerland; 10 Eli Lilly and Company, Indianapolis, IN, United States; 11 Department of Neurology, Indiana University School of Medicine, Indianapolis, IN, United States; 12 MRC Brain Network Dynamics Unit, University of Oxford, Oxford, United Kingdom; 13 Bristol Medical School, University of Bristol, Bristol, United Kingdom; 14 NHS Forth Valley, Scotland, United Kingdom; 15 Re:Cognition Health, Plymouth, Devon, United Kingdom; 16 Re:Cognition Health, Guildford, Surrey, United Kingdom; 17 Department of Neurology, Wessex Neurological Centre, Southampton, United Kingdom; 18 University Hospital Southampton NHS Foundation Trust, Southampton, United Kingdom; 19 Biogen Inc., Cambridge, MA, United States; 20 Department of Anatomy and Neurobiology, Boston University Chobanian & Avedisian School of Medicine, Boston, MA, United States; 21 Neuro TRC, Bristol Myers Squibb, Cambridge, MA, United States

**Keywords:** Alzheimer's disease, at home monitoring, digital biomarkers, feasibility, usability

## Abstract

**Introduction:**

Dementias, including those caused by Alzheimer's Disease (AD), are a leading global cause of death, necessitating improvements in early detection and development of more effective disease-modifying therapies. Well-validated pen-and-paper measures serve as the primary method used for cognitive assessment in research and clinical trials in AD. However, these suffer from rater error, infrequent “snapshot” bias, and have limited sensitivity to early-stage pathology, which presents challenges for measuring efficacy in prevention trials. Recent technological advances offer the potential to measure subtle cognitive changes and account for day-to-day variability through real-world data collection. The Cumulus Neuroscience NeuLogiq® platform (“the platform”), is comprised of a wireless electroencephalography (EEG) headset, tablet-based cognitive tasks based on well-established paradigms which were designed to be user-friendly based on patient panel inputs, third party integrations (mood, speech and sleep assessments) and cloud-based analytics. This platform has demonstrated utility in healthy populations and may enable objective, frequent, and patient-centered disease tracking in AD and other CNS disorders.

**Methods:**

This paper presents findings from a 52-week study involving individuals with mild AD dementia and healthy controls. Analyses focus on usability (e.g., ease of use ratings and reported technical issues) and feasibility (e.g., adherence; withdrawal rates) of using the platform, unsupervised, in the real-world at home setting and exploring how baseline cognitive status and demographic factors impact platform usability.

**Results:**

Longitudinal study data after 12 months offer valuable insights into the feasibility of the platform for patients with mild AD enrolled in long-term studies, such as clinical trials. The participants' high adherence to the protocol underscores the practicality of utilizing the platform in this context. Although participants with dementia reported lower confidence levels (31.6%, N = 18) and encountered some minor technical challenges during the initial home setup (accounting for 16.1% of issues reported in Stage 1), they nonetheless demonstrated strong engagement, achieving an overall adherence rate of 77% across the 52-week study protocol.

**Discussion:**

This demonstrates that even participants with dementia remain able and willing to use the EEG headset and complete tablet-based cognitive tasks over a year, from the comfort of their homes.

## 1 Introduction

Dementias, including those due to Alzheimer's Disease (AD), are the leading cause of death in the UK, second leading cause in the US (1), and the seventh leading cause worldwide (2). Well established tools for measurement of dementia severity and treatment response currently exist and are utilized extensively in clinical trials and research. More specifically, tools such as the Alzheimer's Disease Assessment Scale-Cognitive Subscale (ADAS-Cog) and the Clinical Dementia Rating (CDR) scale are accepted by regulators as primary endpoints in measuring the efficacy of new therapies. Concerns have been raised regarding the sensitivity of the ADAS-Cog to symptoms in earlier phases of disease and its ability to measure reliably and sensitively the smaller changes in cognitive impairment that may occur in the timescale of a clinical trial (3–9). These limitations may contribute to the failure of many clinical trials for novel AD therapies (3, 5, 10, 11). One practical driver of this limited sensitivity is that the ADAS-Cog and CDR are prone to errors of administration and scoring, typically necessitating secondary review with re-scoring and/or central review for multi-site studies (12). The costs and logistical burden of clinician-administered tools also limit the number of times a test can be given and so are vulnerable to day-to-day variation of patient presentation. In combination, these issues substantially increase the requisite scale, duration, and ultimately, cost of clinical trials of disease-modifying therapies in AD, which recently have tended to target early disease.

Recent advances in digital technology have enabled precise, reliable and objective measurement of cognition, behavior and neural function across the disease spectrum, thus potentially improving a) the sensitivity and specificity of diagnosis; b) the ability to detect change due to an intervention, or progression of the disease (13–15); and c) screening for large-N clinical research (e.g., ADNI4 (16, 17)). Specifically, repeated measurement of brain activity and behavior across several days may allow for extraction of more reliable metrics that can overcome day-to-day variability than is possible for in-clinic measures (18, 19) and can be administered in settings more representative of patients' daily lives (20–22). Improvements in measurement reliability can translate directly into statistical power, potentially lowering the sample size required to detect a treatment effect (or lack thereof), and/or reducing the required length of clinical trials in AD (23–25). However, an important pre-requisite for digital technologies intended to be used in this way is demonstrated usability and feasibility in the target population(s) (26) as data collection depends on patients' ability and willingness to engage with the devices and software in question. This is the issue addressed by the current paper.

In response to the opportunity outlined above, the Cumulus Neuroscience NeuLogiq® platform was developed to deliver frequent, at home, longitudinal assessments of neurocognitive function. The platform combines neurophysiological monitoring (EEG) synchronized with tablet-based assessment tools and cognitive tasks (27) and third-party integrations. These integrations include the Dreem sleep headband (28) and applications assessing domains of mood/emotional bias (29) and speech (30).

The main aim of this paper is to examine the feasibility of at home measurement via the platform in a cohort of participants with early stage (mild dementia) Alzheimer's Disease and healthy, age-matched adults in a 52-week longitudinal study. Assessing feasibility of remote, unsupervised measurement tools in Alzheimer's disease (preclinical and manifest) is an area of research which is rapidly developing (14). Previous studies evaluating the feasibility of remote technological measurement tools in a mild dementia or preclinical Alzheimer's disease population over a similar duration (1 year) have reported rates of adherence as a metric of feasibility—with widely varying results. For example, Jutten et al. (31) reported 78% adherence to the requested protocol (mean 11.7 of 15 requested cognitive assessments over 12 months). In comparison, Berron et al. (32) reported that only 62.5% of enrolled participants completed one or more valid cognitive test sessions (of 24 requested sessions over 12 months). More positively, Butler et al. (33) reported that after 12 months, 71% and 54.9% of their MCI participants were still contributing passive (iPhone) and active (monthly CANTAB cognitive test) data respectively to the study after 12 months. In a shorter (8-week) high-demand study with several devices and apps, Muurling et al. (34) observed 63%–95% adherence to the requested protocol depending on the measure and stage of disease (from preclinical to mild-moderate). Using literature to establish a benchmark for acceptable adherence is challenging due to substantial variance in requested engagement and researcher contact (i.e., session reminders) involved in longitudinal remote study participation. The current study represents the first deployment of the platform in patients diagnosed with AD dementia and may be considered a relatively high-engagement and medium-contact protocol by literature standards. Sessions involve setup and use of multiple devices (EEG headset and tablet), a variable testing schedule (as frequently as daily, in the highest-intensity phase, and 69 requested sessions over 12 months), and periodic email reminders outlining the required sessions expected at the start of each new study phase.

A secondary aim of this paper is to investigate whether and how diagnosis and baseline demographic factors (including cognitive ability as scored by the ADAS-Cog, age, level of education and sex) impact the ability to use the platform (incorporating integrated third-party assessments including speech (Winterlight Labs, Canada), mood (P1vital, UK) and also the Dreem sleep headband and app (Dreem, Paris, at the time of study).

Analyses are presented that quantify the usability of the platform, including measures of ease-of-use, withdrawal rate, task duration, EEG data availability, overall levels of adherence, support from study partners and technical issues encountered over a period of 12 months of unsupervised at home use in patients with AD and age-matched healthy volunteers.

## 2 Methods

This was an unblinded, non-randomized, real-world observational study to evaluate the feasibility of the platform to measure neurocognitive function in Alzheimer's disease dementia. The design of the platform and this feasibility study discussed here have been shaped substantially by the Cumulus Pharmaceutical Advisory Group (CPAG), a consortium of industry experts representing ten pharmaceutical companies, who have provided input into decisions related to the design of validation studies (including the current study), task/assessment selection, blood biomarker selection, and will continue to contribute to the ongoing analysis of study data. While CPAG provided guidance on study design, participant eligibility, ethical oversight, and study conduct were governed independently through Research Ethics Committee approval and site Principal Investigator oversight.

The platform comprises (1) a dry sensor, self-setup, wireless electroencephalography (EEG) headset that records brain activity and is an FDA 510(k) cleared/UKCA marked Class 1 medical device, (2) cognitive tasks based on established paradigms presented on a mobile tablet, and (3) cloud-based storage and an automatic processing pipeline which outputs to the NeuLogiq Study Management Hub dashboard for live data integrity checks. All study data were pseudonymised using unique participant identifiers and processed in accordance with UK GDPR and relevant data protection regulations. Data was transferred using encrypted HTTPS protocols and stored within secure cloud infrastructure with controlled, role-based access restricted to authorised study personnel.

The platform integrates third party applications to assess domains of mood/emotional bias (“FERT”) (29) and speech (Winterlight Labs/Cambridge Cognition) (30). Sensitive speech recordings were handled with additional safeguards, and participants were instructed not to disclose personal or identifying information during speech tasks, and any potentially identifying content identified during transcription procedures was redacted prior to any downstream analyses. A second EEG headset, the Dreem sleep headband (“the sleep headband and app”) (28) was introduced for intermittent use overnight to measure patterns and stages of sleep. The platform, the sleep headband and app, and the mood and speech tasks have been tested and validated in healthy populations, in both younger and older adults (27–30, 35), demonstrating that at home EEG and digital assessment is a suitable tool for clinical research with the potential to provide objective, frequent and patient-centered tracking of disease-relevant markers.

As part of the study, participants were asked to use the platform and sleep headband and app at home over a 52-week period. Participants were also required to attend three in-clinic visits: at baseline, at mid-point (week 26), and at the end of the study (week 52). During these visits, they completed pen-and-paper assessments, with optional blood samples collected at weeks 26 and 52.

To minimize error and enhance the detection of subtle disease progression trends, repeated sampling was employed, enabling line-fitting algorithms to improve trend analysis. Multi-stream, multi-sample data of this type can be harnessed with the use of machine-learning techniques to enhance discriminatory power, as demonstrated in studies on aging, dementia, and treatment response (36–38). This approach was designed to reduce variability and improve precision, ultimately streamlining clinical trials by lowering both time and cost in the development of dementia treatments. This analysis is not reported in the current paper, which focuses on usability and feasibility of the platform in this measurement approach.

### 2.1 Participants

A total sample of 119 participants were enrolled across 7 clinical sites in the United Kingdom. Of this sample, 59 patients were diagnosed with early-stage (mild) dementia meeting clinical diagnostic criteria for Alzheimer's Disease (NIA-AA) (39), and 60 were healthy controls. A pragmatic approach was taken to enrolment due to the nature of this non-interventional, observational study. If biomarker evidence of Alzheimer's disease was available to confirm diagnosis, this was desirable and encouraged but not essential for inclusion. Relevant medical history, concomitant medications, and lifestyle factors were collected at screening. Participants with unstable medical or psychiatric conditions, or conditions judged by the investigator likely to interfere with study participation or interpretation of results were excluded. Tables 1, 2 outline the inclusion and exclusion criteria applied at enrolment.

**Table 1:** Study inclusion criteria.

Inclusion criteria		
Both groups	Additional criteria—Dementia group only	Additional criteria—Control group only
Age 50–90 years	Clinical diagnosis of early-stage or mild dementia (ACE-III score >60 and ≤88)	ACE-III score >88
Wi-Fi available in-home	Clinical suspicion of Alzheimer's disease aetiology	No dementia, referral or suspicion of dementia, or other major neurological or psychiatric illness
Fluent in English	Stable dose of medication for 8 weeks (or not on any medication)	
Able to give informed consent	Available study partner to assist if necessary	
In the opinion of the investigator, willing and able (physically and mentally) to take part and adhere to the study visit schedule and other requirements of the study		

**Table 2:** Study exclusion criteria.

Exclusion criteria
Both dementia and control group
A medical condition that in the opinion of the principal investigator would affect participation in the study, or affect the results, including but not limited to: - Schizophrenia - Epilepsy - Brain Tumor
Participation in other research which would, in the opinion of the principal investigator, result in overburden or confound the results.
Barriers to technology integration into their home: e.g., unreliable wireless internet connection.

### 2.2 Recruitment and screening

Site staff approached potentially eligible individuals in person at routine clinic appointments, through telephone or letter of invitation. Control participants were recruited as spouses, family members of the patients, or from existing research volunteer cohorts and the wider community. Informed consent was obtained, and all participants completed screening procedures to confirm suitability for inclusion in the study, including completion of an Addenbrooke's Cognitive Exam III (ACE-III) (40) if not previously completed within 6 months of screening. Participants were considered enrolled in the study once they had signed the consent form and passed screening. Contact details and relevant demographic and health and lifestyle data (including key risk factors for dementia identified by the Lancet Commission) (41) were recorded along with concomitant medications. Due to the pragmatic enrollment approach, there were no restrictions on participants' medication use during the study. The only exception applied to the dementia group, who were required to be on a stable dose of treatment-related medication for eight weeks. Participant personally identifiable information remained at the recruiting clinical sites, and access to this information was limited to authorized site staff and study monitors.

Only participants considered capable of providing informed consent were enrolled in the study. Participant wellbeing and ongoing suitability for participation were monitored throughout by clinical site staff, including consideration of changes in cognitive or physical health status during the study period. Participants were free to withdraw at any time without consequence, and continued participation was reviewed where appropriate. Measures to minimize burden and fatigue included optional home visits and remote support, and splitting assessments across consecutive days if needed. Although a formal distress protocol was not implemented, assessments could be discontinued if participants experienced distress or difficulty engaging with the study procedures or technology.

Site staff discussed the need for a study partner with participants and, if necessary, assisted in identifying one. They also provided study partners with guidance on how they could support the participant throughout the study. The level of support varied based on the participant's individual needs and preferences, including assistance with attending study visits, setting up and using study-related technology at home, and providing reminders for task completion or upcoming visits. Study partners provided practical support only and were explicitly instructed not to complete any tasks assigned to participants. They were given an information sheet detailing their role and were required to provide informed consent for their involvement in the study.

Participants and their study partners, if applicable, were reimbursed for all reasonable travel and/or food and drink expenses incurred when attending the on-site visits. An honorarium of £300 was provided in acknowledgement of a participant's time while taking part in the study.

### 2.3 At home/real world use of the NeuLogiq platform (“the platform”)

Prior to study commencement, a panel of 12 volunteers with lived experience of dementia was convened, consisting of clinical experts (*N* = 6) and people with lived experience as advocates (*N* = 5) or with dementia (*N* = 1). Panel members were involved at the early stages of study design to contribute their perspectives and knowledge, informing task development, study preparation and improve usability and acceptability for older adults and individuals living with dementia.

**Figure 1:**
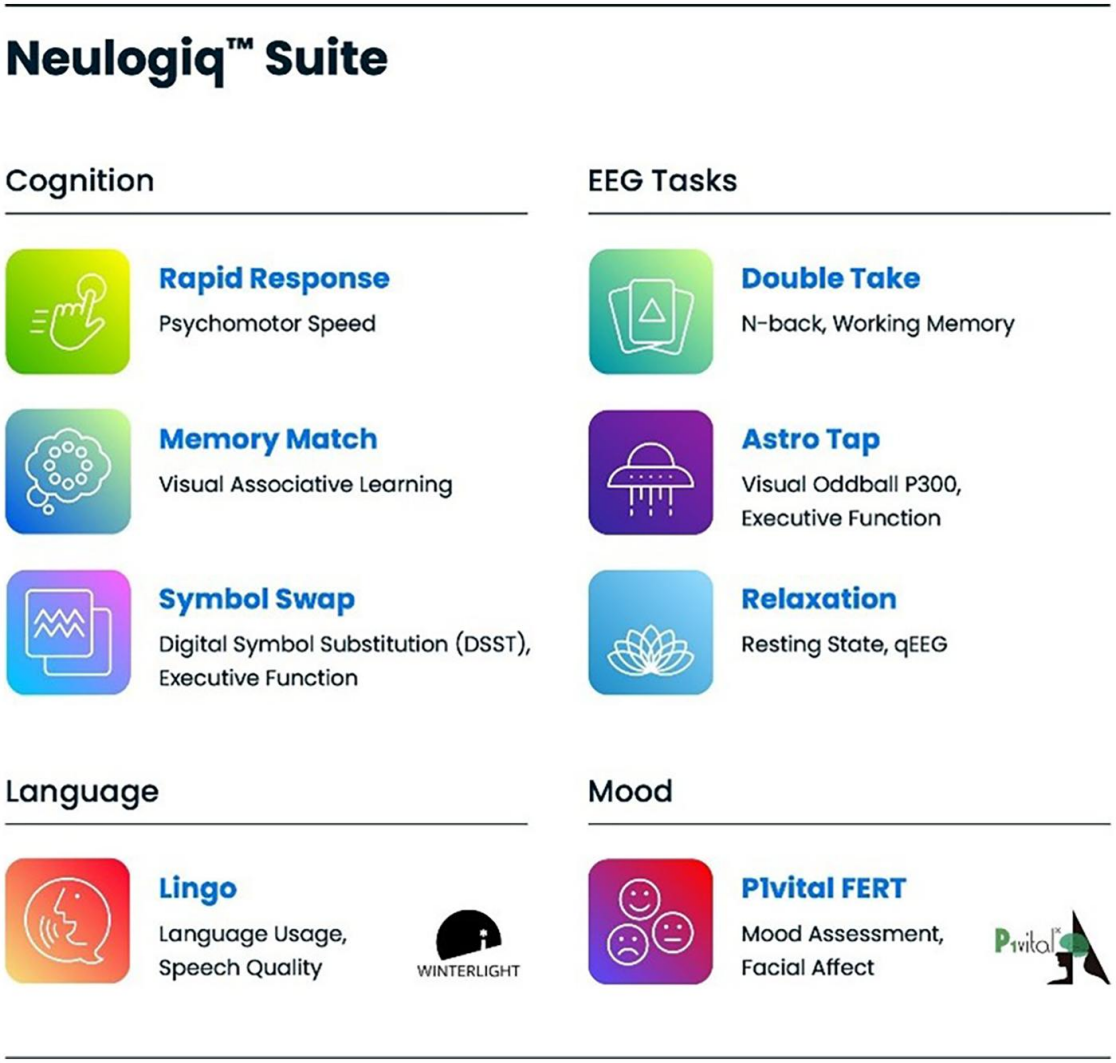
The NeuLogiq tasks completed on the tablet-based app, illustrated as they appear in the app.

Tasks presented on the platform (Figure 1) are based on well-established paradigms from experimental electrophysiology/psychology and cover a range of cognitive functions, lightly gamified with the aim of improving motivation, i.e., enhance attentiveness during testing and prevent boredom over repeated administrations, while maintaining the core cognitive components of the original task (27). Tasks selected for this study included an EEG overlay and were divided across two separate sessions (Session A and Session B) on consecutive days to be completed at home by participants on a Lenovo Android tablet. Table 3 presents the breakdown of tasks completed at home.

**Table 3:** Tasks per session completed by participants at home.

Session A (Day EEG)	Session B (Day EEG)	Session C (Sleep EEG)
“Rapid Response” (Psychomotor task)	“Rapid Response” (Psychomotor task)	Overnight use of Dreem sleep headband^23^
“FERT” (P1vital Products Facial Expression Recognition Task)^24^	“Lingo” (Language task)^25^	
“Relaxation” (Resting state)	“Memory Match” (Episodic memory paired associates test)	
“AstroTap” (Two-stimulus visual oddball)	“Double Take” (N-back)	
	“Symbol swap” (DSST analog)	

Sessions A, B and C were to be completed on consecutive days (with Session C commenced on the evening of Session B), and together, comprised one “cycle”.

Sleep EEG assessment was performed by participants using an additional device—the Dreem sleep headband (28). The headband records, stores and uploads physiological data to a secured Dreem study server through Bluetooth and Wi-Fi connection, where automatic analysis is performed. Overnight recordings were launched by participants through the headband on the night of their second Cumulus session (Session B). Sleep EEG was discontinued five months prior to the conclusion of this study. This decision was necessitated by Dreem entering administration (bankruptcy) in early 2023, after which further data collection was not possible. All enrolled participants were expected to conduct overnight EEG sessions until the point of discontinuation. Digital assessments employed in this study are designed to acquire measurements of cognition, EEG, mood, speech, and sleep. Session completion and data integrity were uploaded to a cloud infrastructure and monitored in near real-time using the NeuLogiq Study Management Hub and Dreem viewer dashboard application, accessed through a web browser window. The cloud infrastructure is hosted by Amazon Web Services (AWS). All systems belong to a Virtual Private Cloud protected by firewalls and with strict access controls. The AWS infrastructure is designed to support safe and compliant storage of patient and other sensitive personal data, under EU-GPDR, UK-GDPR and US (HIPAA) regulations. An electronic Patient Reported Outcome (ePRO) system provided by P1vital assisted with protocol compliance by sending automated emails or text messages to participants, serving as reminders to complete scheduled assessments and sessions.

For technical assistance, the Cumulus Trial Customer Support (TCS) system was used by research personnel at sites to raise issues and queries regarding the Cumulus platform both on behalf of participants and for issues or general questions regarding the technology for themselves.

### 2.4 Standard neuropsychological tests

Standard neuropsychological tests were selected for cohort benchmarking and comparison with platform digital assessments measuring similar constructs (analysis of which is beyond the scope of the current paper). Tests were administered at baseline, mid-point, and end of study and comprised two types: 1) pen-and-paper assessments, administered by site staff in-clinic, and 2) self-administered assessments, which could be completed in-clinic or at home.

The pen-and-paper assessments comprised the Alzheimer's Disease Assessment Scale-cognitive sub-scale (ADAS-Cog13), Coding and Verbal Paired Associates (VPA-I). The ADAS-Cog13 is a validated tool that is used widely in clinical trials as an outcome measure in individuals with AD (42, 43). Coding is a subtest of the Wechsler Adult Intelligence Scale (WAIS-IV) (44), and VPA-I is a subtest of the Wechsler Memory Scale (WMS-IV) (45). The National Adult Reading Test (NART) was an additional pen-and-paper assessment administered at baseline only, and it is used for estimating premorbid intelligence (46).

The self-administered assessments were collected via an ePRO system on a provided Lenovo Android tablet. The patient reported outcomes included the Geriatric Depression Scale (GDS) (47), Apathy Motivation Index (AMI) (48), Sleep Condition Indicator (SCI) (49), Depression Anxiety Stress Scales (DASS) (50), and the Cantril Ladder (CL) measure of life satisfaction (51).

### 2.5 Study procedure

**Figure 2:**
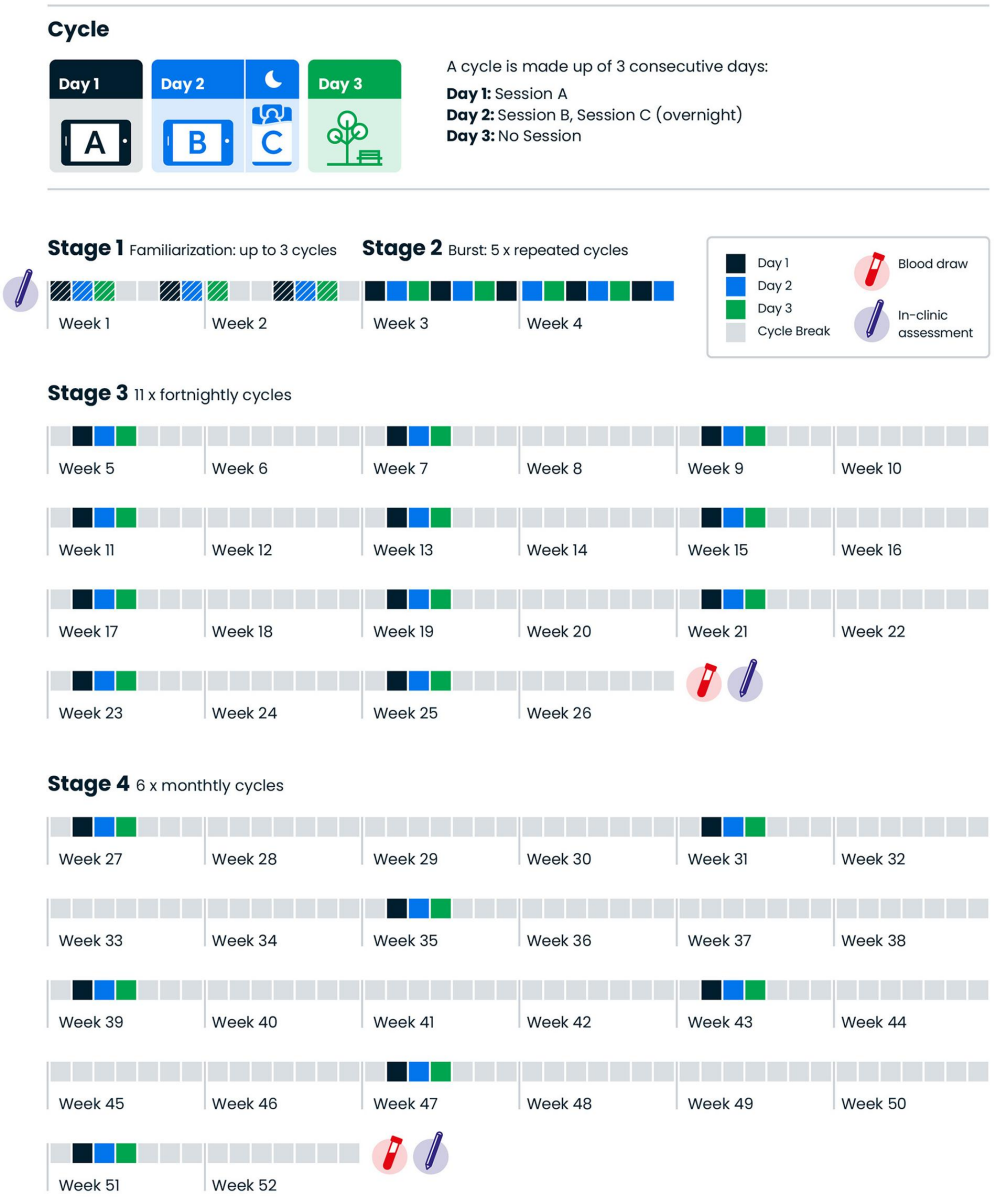
Cumulus assessment schedule.

A schematic of the 52-week long study schedule is detailed in Figure 2. A “platform session” required patients to use the NeuLogiq Platform which is comprised of an EEG headset and tablet-based assessments. Patients captured “overnight sleep recordings” using the Dreem sleep headband and app.

#### 2.5.1 Baseline (in-clinic)

Once the site PI or delegated site team member determined a participant as eligible, participants took part in the supervised baseline assessments: 1) completing the pen-and-paper neuropsychological assessments (ADAS-Cog, Coding, VPA, NART) and 2) undergoing training on the platform prior to at home use. A brief survey was administered by site staff to gauge participants' background technology usage prior to training, and a second survey (Feedback at Baseline survey) was administered via ePRO to capture participants' initial perceptions of the platform, following training. Participants were provided with the option to complete ePRO based assessments (GDS, AMI, SCI, DASS, CL, Technology Feedback at Baseline survey) on-site, or within 1-week of the in-person visit.

#### 2.5.2 Stage 1 (familiarization)

During the first two weeks following training at baseline, participants had the opportunity to familiarize themselves with the technology by completing up to 3 cycles, consisting of 6 platform sessions and 3 overnight sleep recordings. During this period, participants were instructed to complete Week 2 ePRO assessments on the usability of the system (System Usability Scale “SUS”) (52) and a feedback follow up survey (Technology Feedback at Week 2). As participants may have still been exploring the platform during this stage and were not required to complete all available sessions, data from this stage has been excluded from all adherence calculations and models.

#### 2.5.3 Stage 2 (burst)

During Week 3 and 4, sessions ran continuously. Participants were instructed to complete 5 cycles: 10 platform sessions (i.e., Sessions A and B, five times), and 5 overnight sleep recordings (i.e., Session C ten times) in total. This schedule was designed to establish a reliable baseline of more frequent assessments at the beginning of the study and provide access to initial data used to inform and refine the power of the study through planned interim analysis.

#### 2.5.4 Stage 3 (fortnightly)

From weeks 5–26, participants were asked to complete a cycle at approximately 2-week intervals (±1 week, representing 11 total cycles including 22 platform sessions, and 11 overnight sleep recordings in total).

#### 2.5.4 Mid-point (in-clinic; week 26)

Participants were telephoned and invited to return for a mid-point assessment visit. ADAS-Cog13, VPA and Coding were administered by site staff, along with a pen-and-paper based bespoke survey gathering qualitative insights into usability of the platform, sleep headband/app, and ePRO system, in addition to feedback on the study activities. Self-administered questionnaires (GDS, SCI, AMI, DASS, CL, SUS, and Technology Feedback) were completed at home within the ±2-week window of the mid-point assessment. An optional blood sample was also collected on the morning of the visit.

#### 2.5.5 Stage 4 (monthly)

From Weeks 27–52, participants were asked to complete 1 cycle (2 platform sessions, and one overnight sleep recording) at approximately 4-week intervals (±1 week representing 14 platform sessions, and 7 overnight sleep recordings in total).

#### 2.5.6 End of study (in-clinic; week 52)

Participants were invited to return for the final assessment. ADAS-Cog, VPA and Coding were administered by study staff, along with a pen-and-paper based bespoke survey gathering qualitative insights into usability of the platform (NeuLogiq, Dreem, ePRO system) and the study activities. Self-administered questionnaires (GDS, SCI, AMI, DASS, CL, SUS, and Technology Feedback) were done at home within the ±2-week window, and an optional blood sample was collected on the morning of the visit.

#### 2.5.7 Withdrawals

Participants withdrawals were classified by two types, 1) immediate (full) withdrawal, where participants had no more involvement in any study activities, and 2) platform withdrawal, where participants stopped using the platform but continued with other study activities (i.e., clinic visits on month 26 and month 52). For the purposes of this paper, the term “withdrawals” encompasses both categories of withdrawals. Additionally, participants could decide to “opt-out” of completing overnight sleep recordings (Session C) and continue with the rest of the study activities or exercise their right to withdraw fully and have all data removed from the study. The latter option existed but was not exercised by any participant. The withdrawal and opting-out dates were recorded for the adherence analysis.

### 2.6 Data analysis

To gain comprehensive insight into the feasibility of using the platform at home, data analysis was divided into six parts: (1) Participant characteristics, (2) Adherence and Retention, (3) Technology Feedback, (4) Support and Technical Issues, (5) Task duration and (6) proportion of available EEG data from the 52 weeks of data collection. For this paper, the term “group” refers to control and dementia groups.

#### 2.6.1 Participant characteristics

Baseline characteristics were summarized with the mean and the 95% confidence interval (CI) per group if the variable was continuous, and with the count per category if the variable was categorical. Baseline variables included age, sex, education, scores from the pen-and-paper assessments and scores from the self-administered assessments.

Blood biomarker data collected from blood plasma samples at Weeks 26 and 52 were summarized using the mean and 95% confidence interval (CI) for each group. Since blood drawings were optional, not all participants submitted samples. If a participant provided a sample at only one timepoint, that sample was included in the analysis. For participants who provided samples at both time points, the mean of the two samples was calculated to characterize group mean plasma concentrations. These values were not used for longitudinal analyses in the context of this paper. Plasma sample preparation and analyses were conducted by the Biomarker Factory at the University College London (UCL) on five plasma biomarkers of interest: Abeta 40 (A*β*40), Abeta 42 (A*β*42), Glial Fibrillary Acidic Protein (GFAP), phosphorylated tau 217 (pTau217), and Neurofilament light (NFL). The biomarkers were analysed using the Quanterix Simoa Human Neurology 4-Plex E and Alzpath kit according to manufacturer's instructions.

#### 2.6.2 Adherence and retention

Two types of adherence were calculated: the adherence per session type per stage and the pooled adherence. The adherence per session type per stage was calculated by dividing the number of opened sessions of each type in the respective period by the total number of scheduled sessions. The pooled adherence was calculated by dividing the number of opened sessions in the whole study by the total number of scheduled sessions, regardless of session type or stage. Withdrawn participants were included until the date of their withdrawal. Data from those who withdrew immediately following the baseline in-clinic visit or during Stage 1 are excluded from the adherence calculations. Adherence of participants who opted-out of overnight sleep recordings (Session C) was calculated by excluding this session in the number of scheduled sessions from the date participants opted-out. The acquisition of Dreem assets by Beacon Biosignals interrupted the overnight sleep recording data collection from October 2023, therefore after this date, Session C was excluded from the count of number of scheduled sessions. To characterize withdrawals, baseline characteristics of participants who withdrew were compared to those who remained in the study by reporting means and 95% CI of the assessments per group.

To explore variations over time and across session types, as well as potential links between baseline factors and adherence, we explored all combinations of fixed effects from a maximal to a minimal mixed effect model. In the maximal model, the adherence per session type per stage served as the dependent variable, with a three-way interaction involving group, study stage and session type. Age, education, and sex were included as fixed effects, while user was treated as a random effect. The minimal model included the three possible two-way interactions (i.e., the three-way interaction and all covariates were removed). All combinations of covariates and the three-way interaction (from the maximal model to the minimal model, for a total of 16 models) were fitted to the adherence. The Akaike's Information Criteria (AIC) was used to select the model with the least information loss (lowest AIC). The mixed model was fitted using R with the lmerTest package (53), while *post-hoc* tests and estimated marginal means were computed with the emmeans package (54). Normality of residuals was visually checked. Multiple comparisons in the *post-hoc* tests were controlled with the Benjamini-Hochberg false discovery rate method (55) at a level of 0.05. Due to the nature of the familiarization period (Stage 1), this stage was excluded from the mixed models, overall adherence calculations, and subsequent linear models. According to the protocol, participants were encouraged to complete familiarization sessions but were not required to do so.

#### 2.6.3 Technology feedback

Survey data were collected during Baseline sessions prior to training (Background on technology usage), post training (Feedback at Baseline), at week 2, at week 26, and at week 52 (Technology Feedback at week 2, 26, 52). For each survey question, the percentage responses per group and question were calculated, and group differences were assessed with independent *t*-tests. The Benjamini-Hochberg false discovery rate method (55) was applied at a level of 0.05 per survey.

At weeks 2, 26, and 52, participants completed the SUS (52) via ePRO. Scores range from 0 to 100, with scores >70 suggesting good usability, and >80 indicating excellent usability (56). To assess group differences in the SUS' total score, an independent *t*-test was run. The percentages response per group and SUS item are reported.

Given perceptions on new technology may impact the uptake of the platform, two items from the Technology Feedback at Baseline were selected to investigate their association with the pooled adherence. Similarly, the participants' perception of the technology after the whole study could impact the adherence, therefore the association of the SUS score at week 52 with the pooled adherence was investigated. A full model with group, demographic variables (Age, Sex and Education) and the usability variable (“How difficult was it to learn how to use the technology”, “How confident do you feel about using this technology” and the SUS score) was fitted to the pooled adherence of Stages 2–4. Each model was compared to the reduced model which included only the group and demographics variables. An F- test was performed to assess whether the full model was better fit than the reduced model.

#### 2.6.4 Support and technical issues

##### 2.6.4.1 Support from study partners

The number of dementia participants who opted for the assistance of a study partner was broken down by completed participants and withdrawal subgroups. To assess whether the absence of a study partner affected continuation in the study, a Chi squared test was performed. In a follow up analysis, a full model with group, demographic variables (Age, Sex and Education) and a variable coding for the absence/presence of a study partner was fitted to the pooled adherence of Stages 2–4. This full model was compared to the model without the study partner variable. An *F*-test was performed to assess whether the full model was a better fit than the reduced model.

##### 2.6.4.2 Technical issues

During Stages 1–4, user issues were tracked for each affected element (Dreem sleep headband and app, Android tablet, NeuLogiq platform tablet-based tasks and EEG headset) and further subcategorized. Issue subcategories are reported as a % of the total number of issues reported for each product. Similar to the approach used for assessing the presence of a study partner, a comprehensive model was created to evaluate the relationship between pooled adherence and user issues of Stages 2–4. This model included group and demographic variables, along with a binary variable coding for “issue raised/no issue raised” for each participant. This full model was then compared to a version without the raised issue variable, and an *F*-test was conducted to determine if the full model provided a better fit than the reduced model. To investigate associations between the number of issues reported and pooled adherence, Spearman's correlation was calculated for participants who raised issues.

#### 2.6.5 Task duration

The duration for each task was extracted and summed to obtain the total task duration per session. Time between completing tasks during the full session was excluded from this calculation to account for voluntary breaks between tasks in the at home setting. The distribution of task duration was skewed, therefore the median, bootstrapped confidence intervals (CIs) and the Mann Whitney test were used. The Mann Whitney test was applied to assess differences between groups. Only sessions that were complete with full data (behavioral and EEG data) were included in this calculation.

#### 2.6.6 EEG signal availability

The ability to use the platform to record usable EEG in the at home setting was considered by calculating the percentage of time that individual sensors were connected to the scalp (i.e., recording non-saturated data) for each group. The percentage was obtained per user by averaging across sessions per channel. We visualized the mean per channel per group to identify patterns of good connectivity, and calculated the group mean and 95%CI using the mean across channels.

## 3 Results

### 3.1 Participant characteristics

A total of 119 participants were enrolled in the study. Of these, 53 (45%) were female and 66 (55%) were male. The mean age of the study participants was 72.41 (SD 7.04), with ages ranging from 50 to 89 years. Key participant characteristics including selected baseline assessment scores per group and blood plasma biomarkers collected at mid-point and end-of-study are presented in Table 4. A full breakdown of all baseline assessments is provided in Supplementary Table S1.

**Table 4:** Key participant characteristics.

Variable_name	Descriptive	Controls	Dementia
Demographics			
Sex	Males:females, males%–females%	29:31, 48.33%–51.67%	37:22, 62.71%–37.29%
Education	Left formal education before age 16	4 (6.67%)	11 (18.64%)
	Left formal education at age 16	7 (11.67%)	8 (13.56%)
	Left formal education at age 17/18	14 (23.33%)	11 (18.64%)
	To undergraduate degree or equivalent level	25 (41.67%)	14 (23.73%)
	To Master's degree or equivalent level	6 (10.00%)	9 (15.25%)
	To PhD or equivalent level	4 (6.67%)	6 (10.17%)
Age	mean (95%CI), n	71.15 (69.34, 72.96), *n* = 60	73.78 (72.06, 75.50), *n* = 59
Blood biomarkers			
pTau-217 (pg/mL)	Mean (95%CI), n	0.51 (0.41, 0.61), *n* = 47	0.88 (0.71, 1.06), *n* = 47
GFAP (pg/mL)	Mean (95%CI), n	118.97 (100.88, 137.06), *n* = 47	161.82 (139.63, 184.02), *n* = 47
NfL (pg/mL)	Mean (95%CI), n	19.60 (15.21, 24.00), *n* = 47	24.59 (21.68, 27.51), *n* = 46
A*β* 42/40	Mean (95%CI), n	0.06 (0.06, 0.07), *n* = 47	0.06 (0.06, 0.06), *n* = 47
Assessments			
ADASCOG	Mean (95%ci), n	8.88 (7.72, 10.05), *n* = 60	25.07 (23.03, 27.11), *n* = 59
NART	Mean (95%ci), n	41.40 (40.20, 42.60), *n* = 60	36.20 (33.90, 38.50), *n* = 59
ACE III score	Mean (95%ci), n	94.85 (94.07, 95.63), *n* = 60	77.59 (75.78, 79.40), *n* = 59
Verbal paired associates	Mean (95%ci), n	26.35 (24.71, 27.99), *n* = 60	12.20 (10.67, 13.74), *n* = 59
Coding	Mean (95%ci), n	56.72 (53.06, 60.37), *n* = 60	37.78 (34.03, 41.52), *n* = 59
Geriatric depression score	Mean (95%ci), n	1.09 (0.66, 1.52), *n* = 58	3.50 (2.61, 4.39), *n* = 56

### 3.2 Adherence

**Figure 3:**
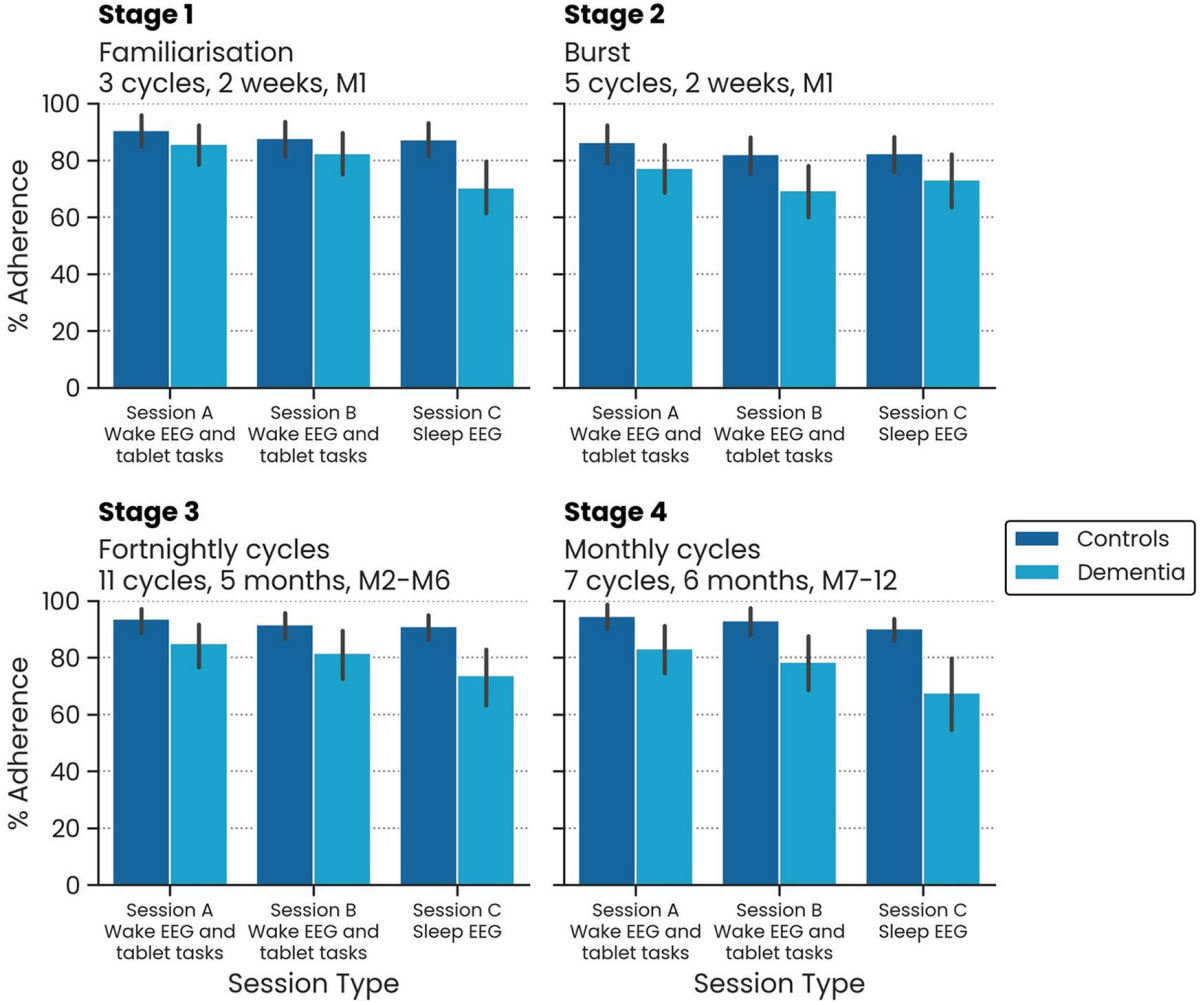
Observed means of adherence across stages and per session type. Error bars depict bootstrapped 95% CI. Stage 1 is included here for completeness.

To investigate group, study stage and session type effects on adherence, mixed effect models were fitted. From Stages 2 to 4, participants in the dementia group completed 2,399 of 2,407 initiated sessions, and controls completed 3,293 of 3,302 initiated sessions. Adherence varied across study stages and participant groups, with pooled adherence to the requested protocol of 77.02% (95% CI 70.0–84.0%) in the patient group and 88.8% (95% CI 84.0–93.6%) in the control group. Figure 3 presents adherence rates categorized by stage and group (see Supplementary Tables S2, S3 for observed means adherence by participant group per stage and observed means adherence by participant group per stage and per session type).

Sixteen models with different combinations of the covariates and the three-way interaction were fitted and ranked by the AIC. The model with the lowest AIC was selected for further analysis and included the three two-pair interactions (Supplementary Tables S4, S5): Diagnosis*Stage+Diagnosis*Session + Stage*Session (the minimal model). Models with the covariates Age, Sex and Education yielded a higher AIC, suggesting that age, sex and education were not significant contributors to adherence, and so were not selected. The selected model revealed significant effects on adherence rates for all main effects and two interactions: a main effect of Diagnosis (F(1, 99.8) = 11.8, *p* = 0.0009), Stage (F(2, 803.3) = 12.8, *p* = 3.3 × 10^−6^), and Session Type (F(2, 802.30) = 19.7, *p* = 4.7 × 10^−9^), and an interaction effect of Diagnosis and Stage (F(2, 803.4) = 5.5, *p* = 0.0043), and Diagnosis and Session Type (F(2, 802.3) = 3.5, *p* = 0.0305).

Post-hoc tests were controlled for multiple comparisons along with the estimated marginal means (EMM) can be found in Supplementary Tables S6, S7. Here we focus on the effects of interest. The dementia group had lower adherence (EMM = 73.8) compared to the control group (EMM = 88.1) (t(99.8) = 3.4, *p_corrected_* = 0.0029). Throughout the study, the adherence increased from Stage 2 (EMM = 77.5) to Stage 3 (EMM = 85.0) (t(801.5) = −5.0, *p_corrected_* = 3.9 × 10^−6^), followed by a decrease from Stage 3 (EMM = 85.0) to Stage 4 (EMM = 80.5) (t(803.5) = 2.9, *p_corrected_ *= 0.0074). Session types yielded different adherence levels, with Session C having lower adherence (EMM = 75.9) compared to Session A (EMM = 85.5) (t(803.5) = 6.3, *p_corrected_* = 8.3 × 10^−9^) and to Session B (EMM = 81.6) (t(803.5) = 3.7, *p_corrected_* = 0.0009).

Group differences were found in the adherence across stages (Group*Stage interaction) and across session types (Group*Session interaction). In the dementia group, adherence declined from Stage 2 (EMM = 71.8) to Stage 4 (EMM = 70.4), while in the control group the adherence increased (EMM = 83.1 in Stage 2 and EMM = 90.6 in Stage 4), (t(805.2) = −2.9, *p_corrected_* = 0.0074. The adherence in Session C was lower than Session A and B in both groups, however, the dementia group showed a larger decrease compared to the control group (EMM = 80.2 vs. 66.5, with a difference of 13.7 for the dementia group, and EMM = 90.8 vs. 85.3, with a difference of 5.5 for the control group), t(803.5) = −2.6, *p_corrected_* = 0.0130).

### 3.3 Retention

**Figure 4:**
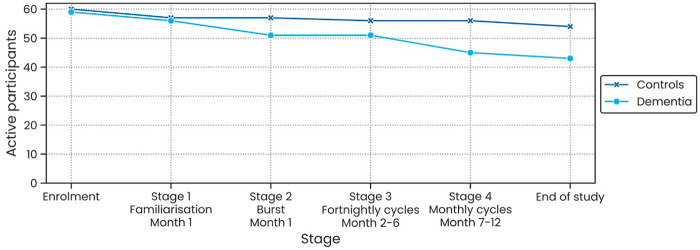
Participant retention rate per study stages for NeuLogiq. Participants are regarded as “Active” here if they had not withdrawn from using the platform in the home. Withdrawals encompass participants who had withdrawn from using the NeuLogiq platform (either as a “full” withdrawal, or “NeuLogiq” withdrawal).

Over the 52-week period, 22 participants (16 Alzheimer's-type Dementia, 6 Controls) decided to withdraw from remote measurement, yielding an attrition rate of 18.5%. Among them, *N* = 18 opted to immediately withdraw from *all* study elements (13 Dementia, 5 Controls), and *N* = 4 (3 Dementia, 1 Control) withdrew only from the technological aspects (Cumulus and overnight EEG). Separately, a total of *N* = 11 (8 Dementia, 3 Controls) opted to discontinue using the sleep headband and app only. Among them, *N* = 3 (Dementia) later decided to withdraw entirely, while the remaining *N* = 8 continued with the other study elements. These 8 participants are not considered withdrawals. Figure 4 shows the number of withdrawals for each group per study stage.

Tables 5, 6 present the reasons for withdrawals and overnight sleep recording opt-outs, and their corresponding percentages per group. The most common reasons for withdrawing were changes in personal circumstances and feeling overwhelmed by the technology and study demands. Participants who decided to opt-out of using the sleep headband and app cited finding the headband uncomfortable and difficult to use as the main reason. Two participants (1 dementia, 1 control) chose not to use the sleep headband and app from the beginning, with no reason provided.

**Table 5:** Reasons and corresponding % for withdrawals.

Reason	Dementia (%)	Control (%)
Change in personal circumstances	50% (8/16)	16.7% (1/6)
Overwhelmed by technology and study demands	43.7% (7/16)	33.3% (2/6)
Headset not suitable	0% (0/16)	16.7% (1/6)
Deceased	0% (0/16)	16.7% (1/6)
No reason provided	6.3% (1/16)	16.7% (1/6)

Percentage per group may not total to 100% due to rounding.

**Table 6:** Reasons and corresponding % for overnight sleep recording discontinuation.

Reason	Dementia (%)	Control (%)
Experiencing adverse event after use	0% (0/7)	25% (1/4)
Headband uncomfortable and difficult to use	85.7% (6/7)	50% (2/4)
No reason provided	14.3% (1/7)	25% (1/4)

Participants in the control group who dropped out were generally older (75.3, 95%CI: 70.2–80.5) and had poorer performance in the ADAS-Cog (13.2, 95%CI: 9.0–17.4) than those who remained in the study (age: 70.7 (95%CI: 68.8–72.6); ADAS-Cog: 8.4 (95%CI: 7.3–9.6)). Withdrawals in the dementia group also tended to be older (76.6 years old, 95% CI: 73.2–80.1), and had lower cognitive ability, as scored by the ADAS-Cog (28.0, 95% CI: 24.3–31.7)), lower pre-morbid IQ as scored by NART (38.4, 95% CI: 35.3–41.6), and associative memory, as scored by the VPA assessment (9.4, 95% CI: 6.7–12.2). Further details of baseline characteristics of withdrawals including aggregated blood biomarkers are presented in Supplementary Table S8.

### 3.4 Technology feedback

#### 3.4.1 Background on technology usage

**Figure 5:**
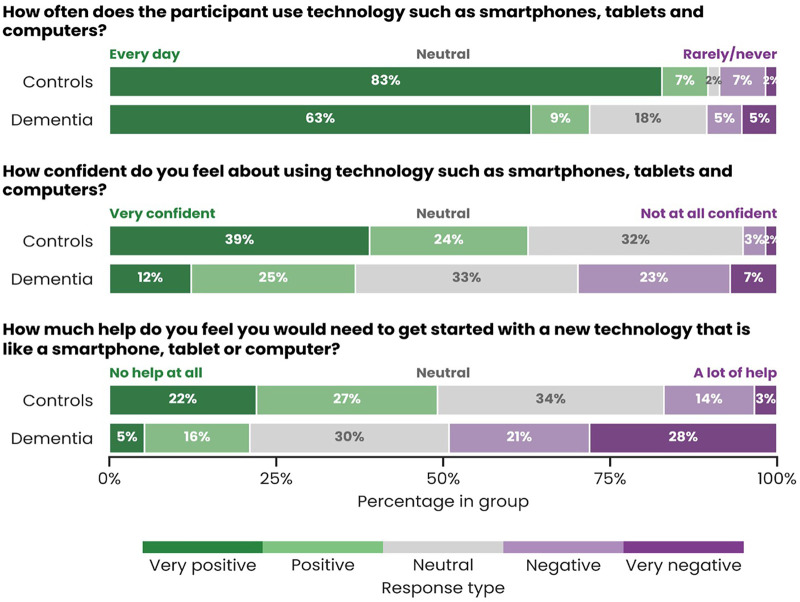
Percentage of responses per group from the background on technology usage survey. Percentages per group may not total 100% due to rounding.

**Figure 6:**
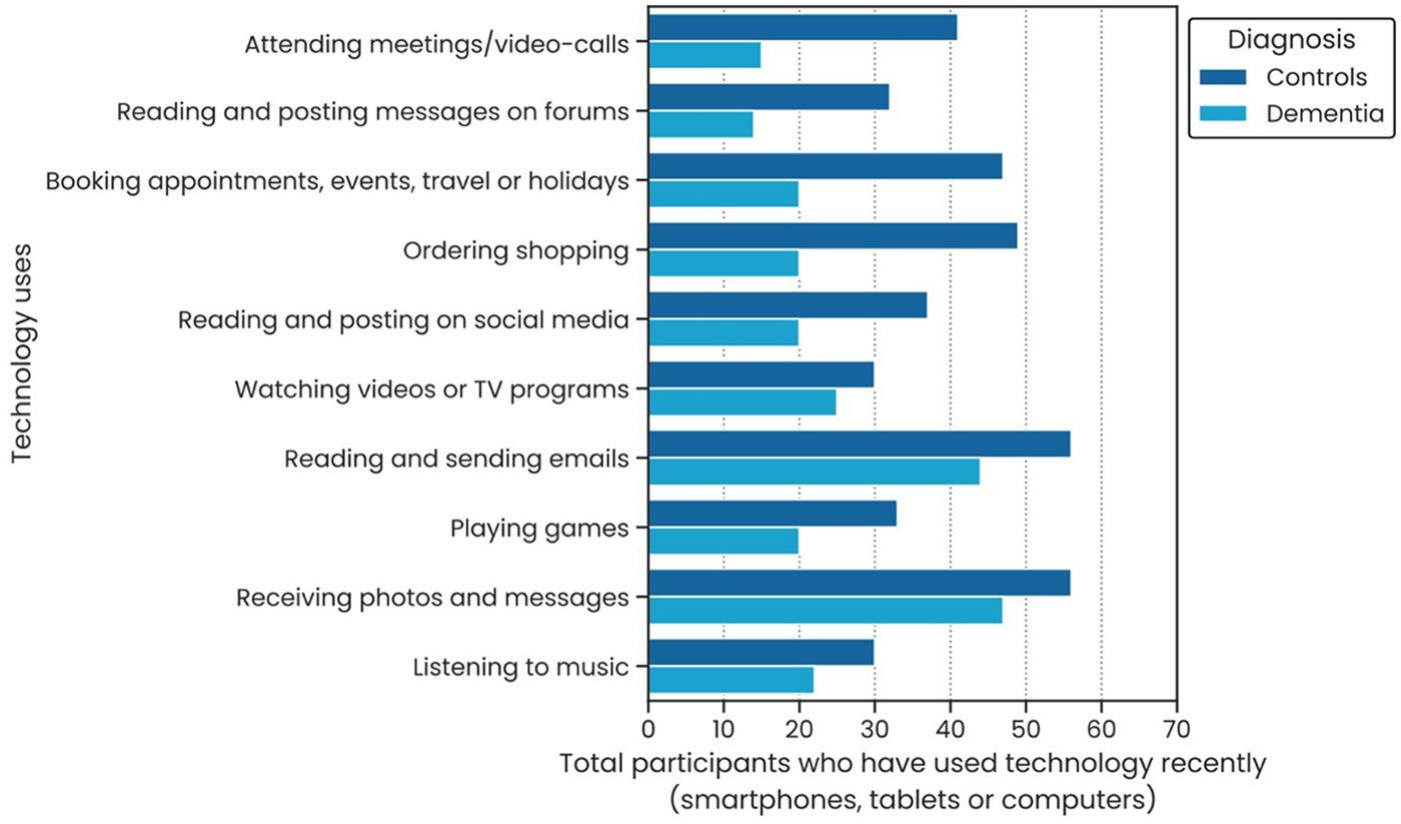
Count of recent uses of technology, where participants could select multiple options.

Prior to training, participants filled out a baseline survey on background technology usage (see Supplementary Table S9). Upon being asked “How confident you are using technology”, controls reported feeling more confident than the dementia group (t(114) = −4.18, *p_corrected_* = 8.52 × 10^−5^, Cohen's d = −0.78), and require less support when using new technology than the dementia group (t(114) = −4.76, *p_corrected_ *= 1.73 × 10^−05^, Cohen's d = −0.88). Most participants used technology, such as smartphones, tablets, and computers every day, with a higher percentage in the control group (82.8%) compared to the dementia group (63.2%) (Figure 5 - with %). When asked to report on the different uses of technology, control participants showed more varied use types compared to the dementia group. Reading and sending emails and receiving photos and messages were the most popular use type among both patients and healthy controls. (Figure 6).

#### 3.4.2 Technology feedback at baseline

**Figure 7:**
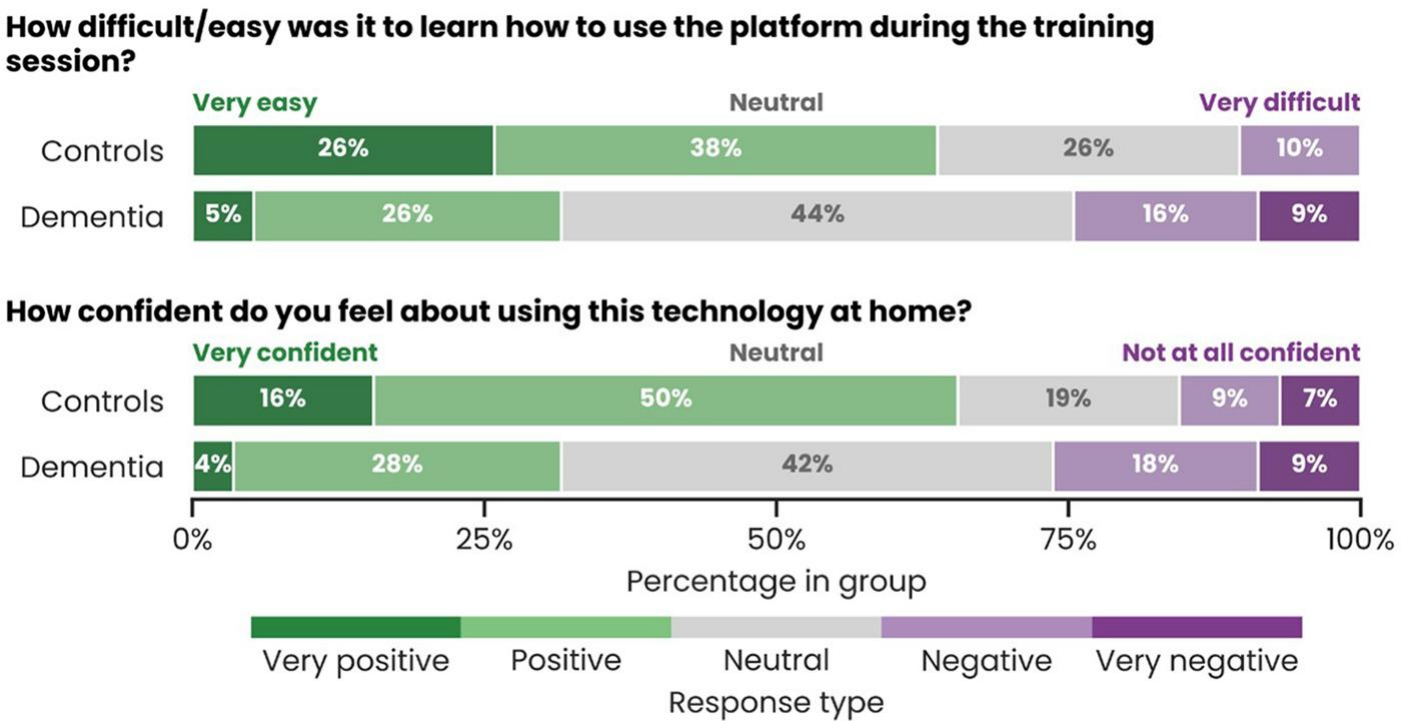
Technology feedback survey at baseline (percentages of responses per group). Percentages per group may not total 100% due to rounding.

Following the technology training at baseline, participants were asked to rate “how difficult was it to learn how to use the technology” and “how confident do you feel about using this platform at home” on the ePRO system. Dementia participants reported more difficulty learning how to use the platform than controls (t(113) = −4.17, *p_corrected_ *= 0.0001, Cohen's d = −0.78), with 8.8% (*N* = 5) of dementia participants providing a rating of 1 (very difficult) compared to *N* = 0 controls. Most control participants reported the platform easy to use (63.8%; *N* = 37; rating> = 4). Dementia participants felt less confident about using the technology than controls (t(113) = −3.05, *p_corrected_ *= 0.003, Cohens' d = −0.57), with controls feeling more confident about using the technology at home (65.5%; *N* = 38) compared to 31.6% (*N* = 18) of dementia participants (rating >=4) (Figure 7). Including the items “How difficult was it to learn how to use the technology” and “How confident do you feel about using this technology” as predictor variables of the pooled adherence was not a better fit than the model with only group and demographics (F(1) = 0.325, *p* = 0.570, and F(1) = 0.150, *p* = 0.669, respectively. Therefore, there was no evidence to conclude that the perceived difficulty and participants' confidence were associated with the pooled adherence. Percentages of responses for all Technology Feedback (including NeuLogiq, Dreem and P1vital ePRO system) at weeks 2, 26 and 52 and accompanying statistics can be found in Supplementary Tables S9, S10.

#### 3.4.3 Technology feedback at week 2, 26 and 52

Following two (Stage 1), 26 (Stage 3) and 52 (Stage 4) weeks use of the technology at home, active participants completed Technology Feedback on ePRO. At week 2, dementia participants reported requiring more support to enable session completion than controls (t(91) = −6.05, *p_corrected_ *= 2.35 × 10^−7^, Cohen's d = −1.27), with 67.9% (*N* = 36) of controls reporting requiring little support (rating >=4) compared to 20% (*N* = 8) of dementia participants. A similar trend was observed following 6 months (t(90) = −5.6, *p_corrected_ *= 1.13 × 10^−6^, Cohen's d = −1.13) and 12 months of use (t(80) = −7.25, *p_corrected_ *= 3.54 × 10^−9^, Cohen's d = −1.53), with 120% increase in dementia participants requiring little or no support to complete sessions at home at week 52 (44.1%), compared to week 2 (20%,), and a 38% increase for controls (67.9% week 2; 93.8% week 52).

No statistical difference was observed between the two groups when rating the ease of use of the platform and the fatigue during sessions (all *p_corrected_ >* 0.55). The platform was considered easy (rating >=4) by 52.8% (*N* = 28) of controls and 20% (*N* = 8) of dementia participants at Stage 1. Ratings for ease of use increased at Week 26 and Week 52, with half of the dementia participants (50%) describing the headset and tablet together easy to use (rating >=4), and 75% of controls at 12 months. At week 2, 69.8% of controls and 55% of dementia participants considered sessions as not tiring (rating >=4) compared to 83.3% of controls and 70.6% of dementia participants with ratings >=4 at week 52. Finally, more than half of the control and dementia group deemed sessions easy to fit into their daily schedule (rating >=4) throughout the study period (Week 2: 60.4% of controls, 60% of participants with dementia; Week 26: 66.7% of controls, 63.2% of participants with dementia; Week 52: 83.3% of controls, 76.5% of participants with dementia).

No group differences were found in the participants' responses to rating sleep quality during the overnight sleep recording sessions. At each stage, more than half of controls reported experiencing a worse sleep with the headband (rating <=2) (Week 2: 51.9%; Week 26: 61.5%; Week 52: 68.1%). A smaller percentage of dementia participants had worse sleep with the headband (Week 2: 34.2%, Week 26: 34.2%; Week 52: 25%). A small percentage of both groups reported better sleep quality at each stage, with 2.1% of controls and 6.2% of dementia participants rating a score >=4 following the 52-weeks.

### 3.5 System usability scale (SUS)

**Figure 8:**
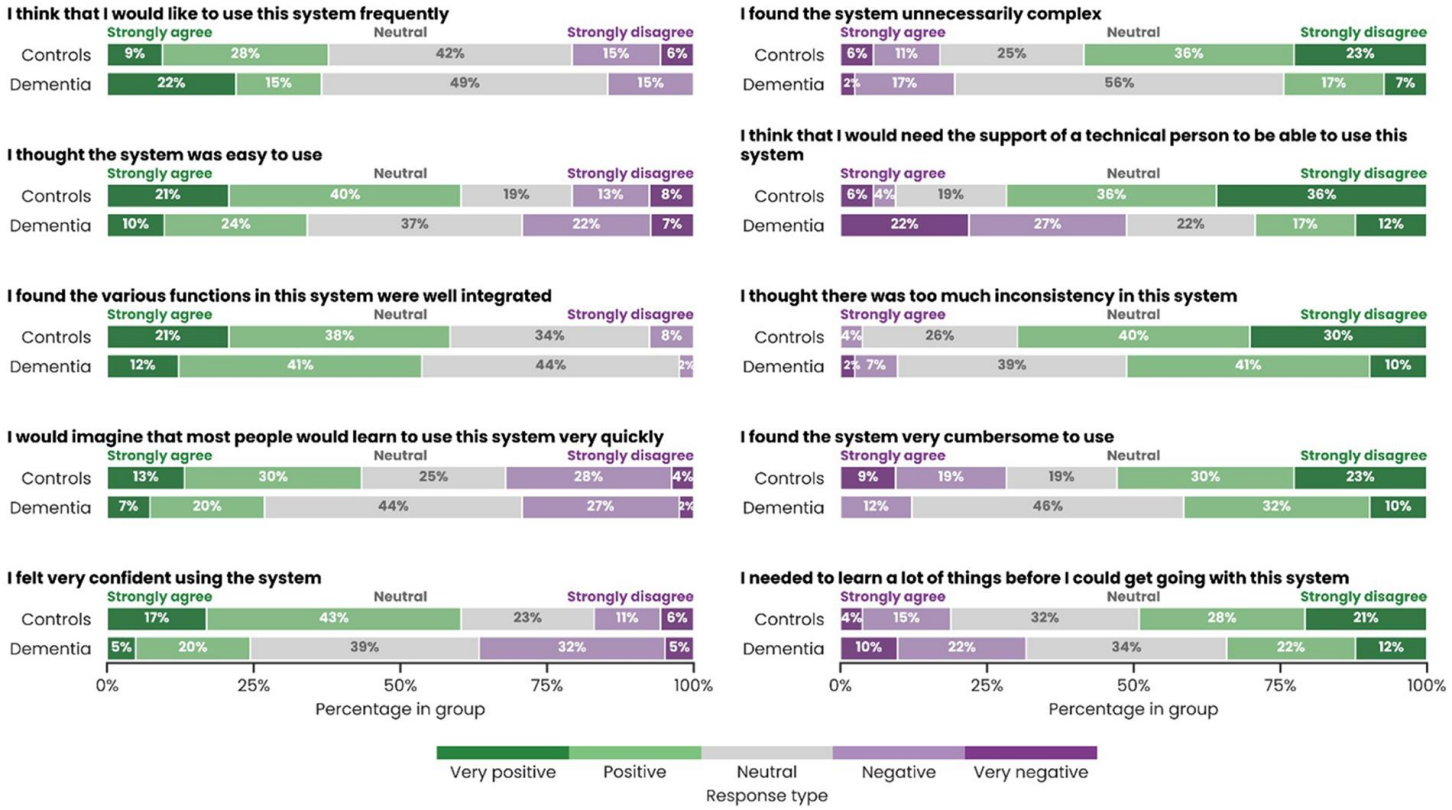
Percentages of responses per group from SUS at Week 2. See Supplementary Figures S1, S2 for Week 26 and Week 52 responses. Percentages per group may not total 100% due to rounding.

Of the 117 participants who were enrolled onto the Cumulus platform, *N* = 93 (40 Dementia, 53 Controls) completed the SUS at Week 2 (Figure 8), *N* = 92 (38 Dementia, 54 Controls) at Week 26 (Supplementary Figure S1), and *N* = 83 (34 Dementia, 49 Controls) at Week 52 (Supplementary Figure S2). These figures reflect the number of participants active by the end of the study who managed to successfully complete the unsupervised assessments at home. An independent samples t-test revealed a statistically significant difference between group ratings at Week 2 (t(92) = ^−^2.62, *p_corrected_* = 0.015 Cohen's d = −0.56), with controls rating the system's usability higher (*M* = 63.8, 95% CI 58.6–69.1, *n* = 53) than dementia participants (M = 54.5, 95%CI 50.3–58.6, *n* = 41) (Table 7). Including the SUS score at week 52 as a predictor variable of the pooled adherence was not a better fit than the model with only group and demographics variables (F(1) = 1.320, *p* = 0.254). Therefore, there was no evidence to conclude that the SUS score at week 52 was associated with pooled adherence.

**Table 7:** System usability scores (SUS) at week 2, 26, and 52.

Timepoint	Dementia [mean (95%CI), *n*]	Controls [mean (95%CI), *n*]	*t* statistic	*p*-value	Corrected *p* value	df	Cohens d
Week 2	54.45 (50.34, 58.56), *n* = 41	63.82 (58.56, 69.09), *n* = 53	−2.62	0.01	0.015	92	−0.56
Week 26	62.24 (54.71, 69.76), *n* = 38	71.81 (67.34, 76.27), *n* = 54	−2.27	0.025	0.025	90	−0.47
Week 52	58.31 (51.53, 65.09), *n* = 34	74.44 (70.06, 78.81), *n* = 49	−4.1	9.72 × 10^−5^	0.0003	81	−0.89

All three tests survived correction for multiple comparisons.

Following the familiarization period, 60.37% of controls found the system easy to use (rating >=4) compared to 32.65% dementia participants. After 26 weeks of use, there was a 20% increase in ease of use (rating >=4) for controls (72%), and a 75% increase for dementia participants (56%). Week 52 showed a decline in ratings for ease of use for dementia participants (52%) from Week 26 scores, however a 59% increase from ratings in Week 2 was observed.

Similarly, most controls (60.38%) felt confident using the system (rating >=4) compared to 25% of dementia participants. Although only a small number of the dementia group felt confident and found the system easy to use, more than half of participants in both groups found the various functions in the system well integrated (52.5% of participants with dementia; 58.49% of controls) and consistent (52.5% of participants with dementia; 69.81% of controls) (rating >=4) at week 2. By week 52, participants felt more confident in using the system (47% of participants with Dementia, 82% of controls) and believed they would not need the support of a technical person to be able to use the system (53% of participants with dementia, 81% of controls). However, there was a decline in system integration ratings among dementia participants (41%), while ratings for controls remained unchanged (58%).

### 3.6 Support and technical issues

#### 3.6.1 Support from study partners

Of the 59 dementia participants enrolled in the study, 21 (35.6%) opted to receive support from a study partner. From the group of withdrawn dementia participants (*n* = 16), 4 participants (25%) opted for a study partner, compared to 17 (40%) of active dementia participants (*N* = 43). A Chi squared test in the contingency table showed that there was no evidence to conclude there is an effect of study partner presence on the decision to withdraw or remain in the study (Chi(1) = 0.534, *p* = 0.46). In a follow-up analysis, the association between study partner presence and pooled adherence of Stages 2–4 was inspected with a linear model. The model that included the study partner variable did not fit better than the model without (F(1) = 2.59, *p* = 0.1148), indicating no evidence to conclude that having a study partner had an effect on adherence in the dementia group.

#### 3.6.2 Technical support

A total of 99 issues were reported in Stages 1–4 of the study on behalf of participants. Of the 99 issues reported, 51 of these were reported on behalf of dementia participants (52%) and 48 on behalf of controls (48%). 25 participants raised more than one issue at home (*N* = 13 dementia, *N* = 12 controls).

**Figure 9:**
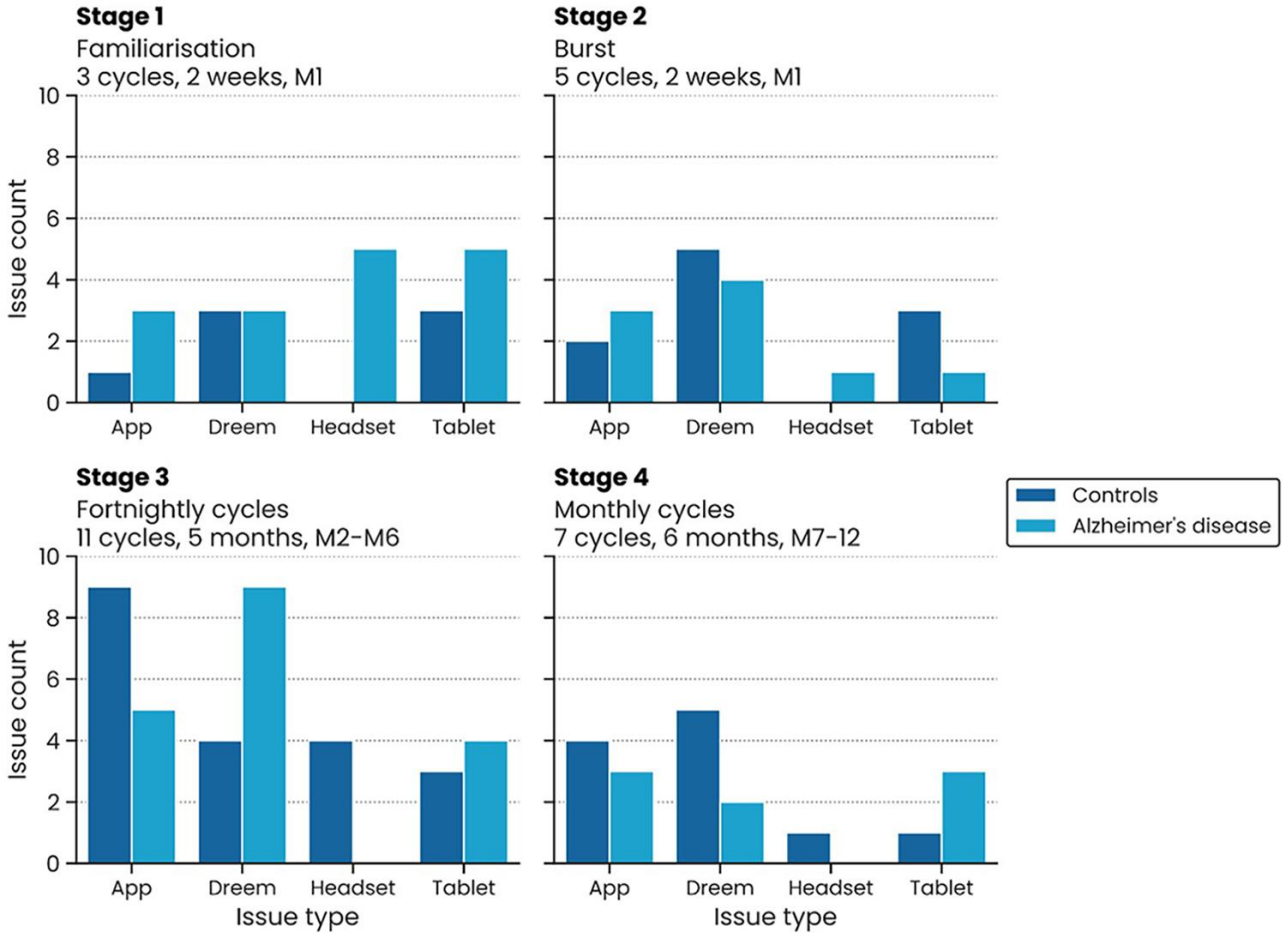
Types of user support issues per study stage per group.

There were 23 reported issues in Stage 1, and 19 issues in Stage 2. There were 16 issues in the dementia group and 7 issues in the control group during Stage 1. In Stage 2, 9 issues were raised on behalf of dementia participants, and 10 issues for controls. There were 38 reported issues in Stage 3, and 19 issues in Stage 4 (Figure 9). There were 18 issues in the dementia group and 20 issues in the control group during Stage 3. In Stage 4, 8 issues were raised on behalf of dementia participants, and 11 issues for controls.

Table 8 shows a breakdown of the issues encountered by participants in Stages 1–4.

**Table 8:** User issue summary.

Product Affected	Total % of issues reported	Issue Summary	Number	% per issue reported
Dreem	35.4%	Faulty Headband	11	31.4%
		Uploading issues	11	31.4%
		Issues launching recordings	6	17.1%
		Software Issues	3	8.6%
		Confusion around Dreem scheduling	2	5.7%
		Headband Fit	1	2.9%
		Battery Issue	1	2.9%
		**Total**	**35**	**100%**
NeuLogiq Application	30.3%	Misunderstanding of scheduling	13	43.3%
		Unexpected task behavior	6	20.0%
		Upload issues	5	16.7%
		Forgotten credentials	5	16.7%
		Mobile App update interference	1	3.3%
		**Total**	**30**	**100%**
Android Tablet	23.2%	Activating unnecessary Android features (e.g., safe mode, screen reader)	12	52.2%
		Screen sensitivity	6	26.1%
		Wi-Fi issues	2	8.7%
		Tablet update interference	1	4.3%
		Battery issues	1	4.3%
		Forgotten pin	1	4.3%
		**Total**	**23**	**100%**
NeuLogiq Headset	11.1%	Battery issues	4	36.4%
		Issues passing through signal sensor check	4	36.4%
		Sizing and Fit	2	18.2%
		Faulty headset	1	9.1%
		**Total**	**11**	**100%**

Bold values indicate the total number of issues reported for each product category, with the corresponding percentage breakdown shown for each issue type within that category.

The association between raised user support issues and pooled adherence of Stages 2–4 was inspected with a linear model. The model that included the binary variable of support issues did not fit better than the model without (F(1) = 1.28, *p* = 0.2608) The subset of participants who raised support issues (*N* = 49) were included in a follow-up analysis, where Spearman's correlation between the adherence and the number of support issues was calculated. No significant correlation was found (rho = 0.15, *p* *=* 0.2948).

### 3.7 Task duration per session

**Figure 10:**
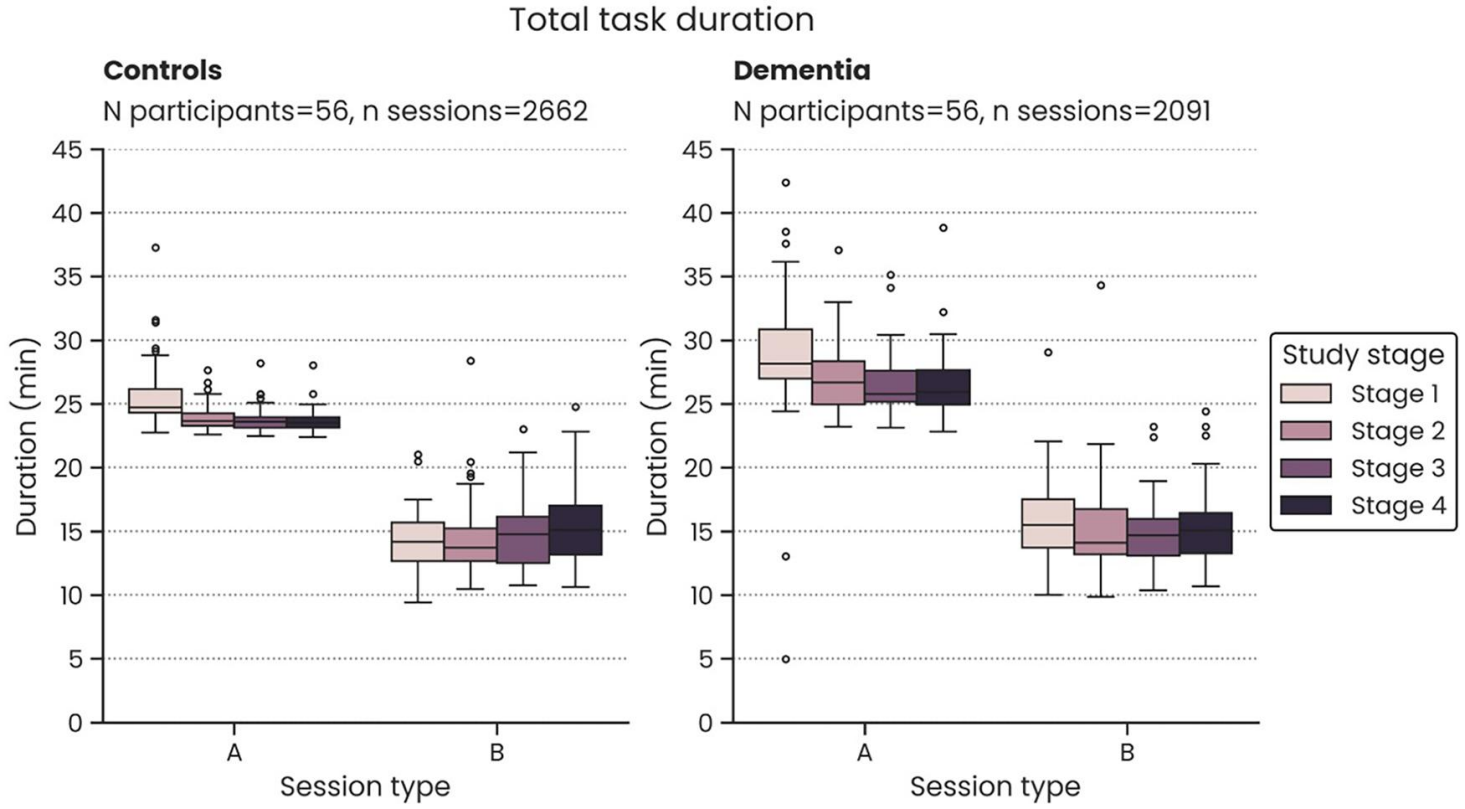
Median task duration per session for participants across different study stages and cohorts. The duration for each task within a session was extracted and summed to obtain the total task duration per session. Time between completing individual tasks within each session was excluded from this calculation to account for voluntary breaks between tasks in the at home setting.

The task duration was significantly longer in the dementia group compared to controls in Session A (Mann Whitney stat = 2,803, p_corrected_ = 1.9 × 10^−13^) but not for session B (Mann Whitney stat = 1,725.5, *p*_corrected_ = 0.28). In Session A, control participants had a median of 23.65 min (95%CI 23.59–24.09) and dementia participants had a median of 26.76 min (95%CI 26.42–28.28) task duration. In Session B, control participants had a median of 14.45 min (95%CI 14.08–15.45) and dementia participants had a median of 14.83 min (95%CI 14.59–16.37) (Figure 10).

### 3.8 EEG signal availability

**Figure 11:**
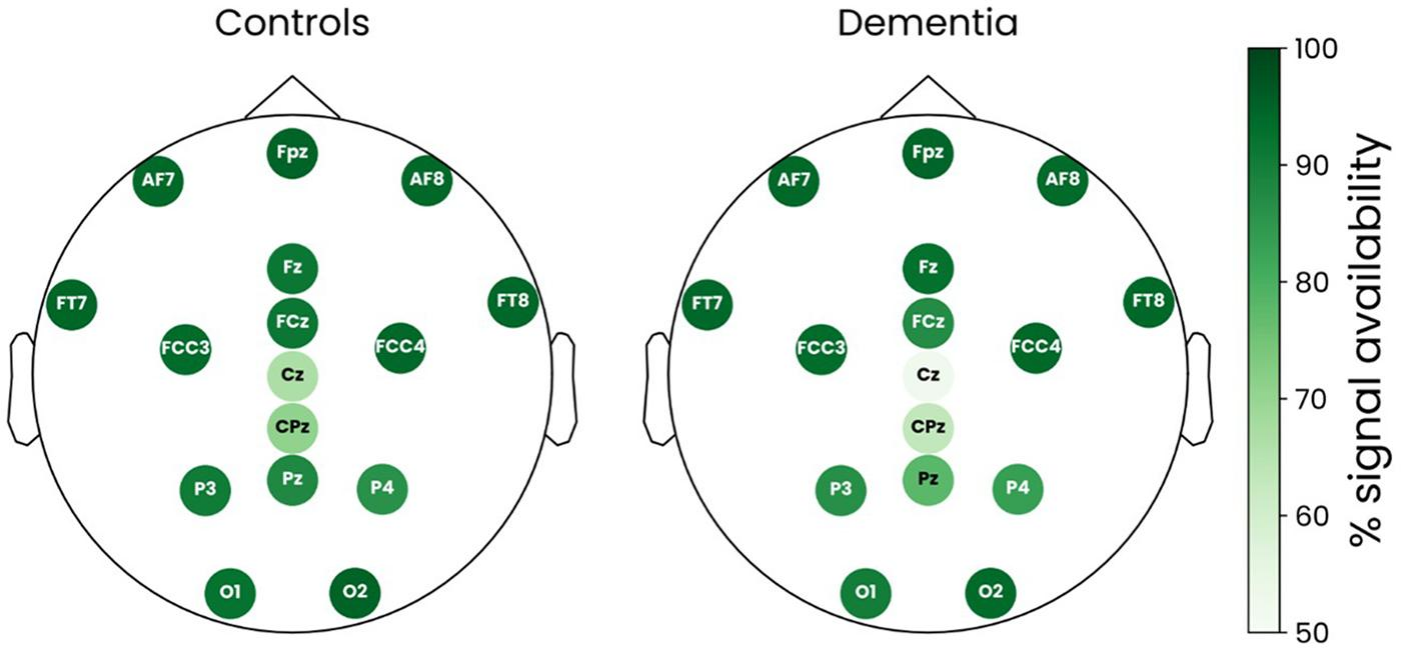
Percentage of time each channel recorded available data, shown per group.

On average, the dementia group had a lower percentage of EEG signal availability (*n* = 58, mean = 86.73%, 95%CI: 84.67–88.79%) than the control group (*n* = 58, mean = 89.65%, 95%CI: 87.57–91.72%). The signal availability percentage was lower in the Cz and CPz channels in the dementia group compared to controls (Figure 11).

## 4 Discussion

### 4.1 Principal findings

This longitudinal study confirms that the platform is feasible for unsupervised use at home by participants with mild dementia.

After a single in-clinic training session, both participants with dementia and age-matched healthy controls were able to use the platform and the sleep headband and app at home without the assistance of medical staff or technicians. Both groups exhibited high adherence to the protocol over 52 weeks (88.8% for controls; 77.02% for dementia participants) relative to other reports of at home monitoring in older populations (∼55%) (57, 58), and cognitively unimpaired but AD biomarker-positive populations (78%) (31). Comparing adherence to previous research to judge the success of a digital platform (in terms of feasibility) is useful albeit challenging due to the lack of an equivalent combination of technology, population and level of participant-researcher interaction (i.e., reminders to complete assessments). The most similar previous study in terms of these factors is a 3-month Cumulus feasibility study involving healthy older adults, demonstrating a mean adherence of 89.6% in the oldest participant group (aged 40 to 79yrs) (27). The current study lasted significantly longer (12 months) and included patients with mild dementia, who reported greater difficulty using the technology compared to the control group. The lower total number of initiated sessions observed in the dementia group should also be interpreted in the context of higher withdrawal rates over the study period, which reduced the total number of expected sessions within that cohort over time. Participants could complete up to 78 sessions across the study period (52 platform sessions, and 26 overnight sleep recordings), and among the participants that remained enrolled for the full study duration, this corresponded to a theoretical maximum of approximately 3,354 sessions in the dementia group, compared to 4,212 sessions in the group. Therefore, the lower number of initiated sessions in the dementia group (2,407 vs. 3,302) was influenced in part by reduced participant retention, rather than solely reflecting reduced engagement among active participants. In addition, participants with dementia may experience challenges associated with cognitive impairment and functional symptoms that may affect engagement with digital technologies. For example, cognitive and functional decline combined with diverse behavioral changes, such as mood changes, agitation, and apathy, are known to manifest with increased severity over the disease course (59). Life events, time constraints or competing priorities also may also pose challenges for individuals to maintain their commitment over a period, with evidence showing even for relatively young and healthy participants, 15% of assessments can be missed in two-week cognitive assessment protocols (60).

It may be useful to consider adherence to this protocol in the context of what arrangement might be required in a typical AD clinical trial, which the platform is designed to support. The classic model of measurement in Alzheimer's disease is snapshot clinic visits with large gaps (6–12 months) between assessments, in part due to the slow pace of disease progression. Hassenstab et al. (24) outlined the potential benefits of digital “burst measurement” in AD clinical trials, which could potentially be administered concurrently with the traditional measurement timepoints using a digital platform. Stage 2 of the protocol, which involved near-daily assessments over two weeks, closely resembles a burst measurement. During this stage, adherence was 82.11% for controls and 69.99% for patients, respectively. An optimal digitally enhanced trial protocol might involve periodic (e.g., annual or biannually) burst periods of intensive measurement. However, in the current study we see a clear pattern of adherence improving with *less* frequent assessments over time. Additional studies are needed to determine the optimal frequency, duration and structure of burst measurement paradigms (e.g., near-daily assessments over one week, repeated quarterly) that best balance participant burden, adherence and sensitivity to disease related changes. Regarding the current study and protocol arrangement, overall adherence statistics demonstrate that it is feasible to collect digital biomarkers in the home from mild dementia patients using the platform. Additional analyses are currently underway to evaluate the sensitivity of each digital measure and modality for distinguishing disease state at baseline and detecting longitudinal change over time. Whether combinations of digital measures provide greater sensitivity than individual modalities alone remains an open question. The current study was not specifically designed or powered to evaluate multimodal composite models, and the large number of possible combinations introduces risks of overfitting and limited generalizability. Future work therefore will be required to determine whether multimodal approaches provide meaningful advantages over individual measures, particularly in applications such as clinical trial screening or patient stratification (61).

To understand where improvements might be made, detailed information was collected on withdrawals and technical issues throughout the study. Around 18% of participants withdrew from the at home measurements, and approximately half of them cited technology burden as a reason. When examining technical issues, the three most common issues reported related to participants missing the assigned time windows to complete their sessions, participants involuntarily activating unnecessary Android features, and technical issues with the sleep headband and/or app. These results suggest that improvements can be made to session reminders and specific technical aspects of the technologies. More than a third of the technical issues reported were related to the use of the sleep headband and/or app, with problems uploading recordings and faulty headbands being the most often reported issues. Some participants (<9%) also opted to stop using the sleep headband specifically, while continuing to use the platform for cognition and wake-EEG measurement. Discomfort and difficulty in using the sleep headband were cited as main reasons for opting out. These observations are congruent with a recent study from De Anda-Duran and colleagues (62) who reported low level of technology acceptance, with 60% of participants declining to use the sleep headband and app when asked to actively opt-in and similarly citing problems of discomfort and/or effect on sleep as the main reason to stop using it. Furthermore, participants in both groups who continued using both sets of technology throughout the study showed higher adherence to the platform sessions compared to the overnight sleep recording sessions, with poorer sleep quality on nights when wearing the sleep headband, as reported by controls through Technology Feedback. However, it is worth noting that participants in this study were required to interact with a second user interface that differed in terms of UX and interaction types, which may have resulted in a higher level of burden and difficulty. This issue is commonly linked to less seamless integration of technologies (63), thus greater integration of sleep technology into the platform might improve usability.

Those who fully withdrew from at home measurement in both patient and control groups demonstrated a pattern of older age and greater cognitive impairment, on average. This finding is unsurprising, given that the decline in executive function and working memory associated with dementia has been reported to impede usage of technological devices (64). In addition, there was no evidence found for the effect of the presence of a study partner on withdrawal or associated with protocol adherence. Similarly, reporting technical issues, the perceived difficulty of using the new technology and the usability score were not associated with adherence, though technology burden was cited as one of the main reasons for withdrawing. Literature on e-health and digital technologies imply a common belief is that older populations will struggle with digital technologies due to unfamiliarity with smart devices or frustration if the technology malfunctions, leading to decreased participation and higher attrition rates in typical research or clinical settings (65–67). In summary, it appears that experiencing a greater technical burden *per se* seems to affect the feasibility of this approach in dementia, rather than availability of support. This underscores the need for careful, accessible design of technology for use by older and cognitively impaired participants, and ideally, full integration of discrete system elements under a single user interface.

Controls rated the usability of the system as higher than dementia patients across all three timepoints, reflecting the fact that the system usability scale (SUS) is not a pure measure of the system itself, but also the capacities of the user group. Neurological changes associated with disease can make interaction with technology more difficult. The SUS does not suggest hard score cutoffs for usability, but some guidance can be found in the literature. For example, Bangor et al. (68) reported 69.7 as the mean score across 206 published studies. Control participants in the current study rated the system more highly than this value at the mid-point and endpoint of the study, but lower at the beginning, suggesting the presence of familiarity or learning curve. Mean scores in the dementia group were lower than the literature average across all three timepoints (range 54.45–62.24), again with the lowest score at the outset of the study. Consistent with literature (69, 70), participants with dementia felt less confident about using the technology yet adhered well and found the system to be well-integrated and consistent. This aligns with a recent systematic review by Contreras-Somoza et al. (71), which found that MCI and mild dementia patients do typically show the ability to engage with digital cognitive tests but may simply take more time to become familiar with new systems and require more support. Though no statistical relationship was found between the presence of a study partner and adherence, this hints that the involvement and support of study partners (used as required in the current study) may be important in enabling session quality, as opposed to quantity. This interpretation is borne out to some extent by the results from the Technology Feedback questionnaires, in which patients were significantly more likely than controls to report that they needed support to perform sessions at home. It may be that those who needed support from a study partner were generally those who opted to have it.

Although a formal standalone measure of technology acceptability was not administered, acceptability was evaluated indirectly through longitudinal adherence, retention, usability ratings, participant feedback, and perceived burden measures. Interestingly, despite reporting lower usability overall, more than half of participants in both groups reported that the platform sessions were not tiring and were easy to fit into their daily schedule. A possible explanation for these observations is that the at home sessions were considerably shorter (e.g., <30 min) than typical clinical trial visits that may last for several hours. This demonstrates the ease of which digital health technologies assessing cognitive domains can be adapted into the daily routine of individuals living with dementia, relative to attending lengthy in-clinic visits to complete pen-and-paper based assessments, that are known to be fatiguing for those with cognitive impairments (72).

Additional reports of usability through the feedback surveys showed control participants were more likely to report poorer sleep while wearing the sleep headband than dementia participants. While unexpected, these findings may be due to reporting bias, where control participants have a better understanding of the extent of their sleep disruption and were therefore more accurate in reporting it. Sleep quality and depth are already known to be disrupted in AD dementia (73), therefore the addition of a headband during sleep may not add further disruption.Modest differences in task duration between the dementia and control groups in both Session A (3 min) and Session B (1 min) were observed, highlighting the impact of dementia on task performance. The extended task duration in the dementia group is consistent with their cognitive impairments and not so severe as to undermine feasibility of the assessments in the dementia group. These findings align with previous research indicating that individuals with lower cognitive ability may take longer to process cognitive tasks (74). Such differences between groups emphasize the potential richness and sensitivity of digital biomarkers, particularly in the home setting. Traditional clinical assessments may overlook subtle changes in daily cognitive performance, such as task completion time, which is often not measured and can be influenced by the rater's pace. In contrast, frequent at home task completion allows for the precise capture and quantification of this data over time, facilitating the establishment of a stable baseline and the detection of meaningful change. Although completion of digital assessments may take longer to complete than some traditional clinical assessments, completion of these digital assessments in the home setting enables a safe, familiar environment for patients and may be more acceptable to patients and families given the time taken to travel to the clinic, and the stress of the “white coat effect” while testing (75).

Finally, differences in percentages of EEG data availability were evident between the patient and control groups (particularly at Cz and CPz), and may be attributed, in part, to the manual dexterity and adjustment required for EEG sensor placement. Fine motor function challenges in Alzheimer's disease and cognitively impaired individuals have long been acknowledged, with research indicating a strong correlation between manual dexterity deficits, apraxia and dementia severity (76, 77). As a result, adjustments were made in the later stages of the study, introducing enhancements such as optional chin bands (to help sensors on the top of the head to make contact). These modifications aimed to address difficulties associated with sensor placement, enhance participant comfort, and improve data quality. The specific impact of these changes, however, is yet to be determined. Another explanation for the topographical pattern of missing data (centro-parietal focus) could be found in the design and affordances of the headset itself. Head shapes can vary substantially, and it is possible that new adjustment mechanisms, ergonomic factors or instructions could enhance user placement/fit of the headset such that electrodes make better contact with the centro-parietal scalp. These methodological refinements reflect an ongoing effort to optimize EEG data collection in older populations, considering factors such as manual dexterity and adjustment challenges. Further research should delve into the relationship between EEG data availability and cognitive performance, considering the potential mitigating effects of these methodological improvements.

Given the differences in data availability across groups outlined here, future interventional clinical trials employing this methodology (or similar) should be designed such that differences between participant groups in terms of cognitive impairment (i.e., either a) at baseline or b) after long-term treatment which preserves function in the active group but not the placebo group) do not lead to confounding differences in participant adherence or data quality. Active monitoring of incoming data, and periodic quality assurance checks will help to mitigate this.

### 4.2 Limitations

Technical problems occasionally arose within the first 4 weeks, though this did not affect adherence. Measures were put in place to prevent further technical issues from recurring throughout the study, such as de-activating unnecessary android shortcut features such as “safe mode”, improving the headset battery functionality via a firmware update, and updating the participant instruction manual with more detailed guidance for using the sleep headband and app.

The discontinuation of the Dreem device during the study following the company entering administration also highlights the operational risks associated with reliance on third-party technologies in longitudinal digital biomarker studies. While opportunities to mitigate unexpected third-party failure during an active study may be limited, the modular design of the Cumulus platform allows individual software and hardware components to be replaced as technologies evolve, supporting continuity of measurement domains such as sleep physiology, mood, speech, or activity, provided alternative technologies demonstrate appropriate validation, usability, and data quality.

A further limitation of the study is that not all participants completed self-administered assessments via ePRO at home, with more participants from the control group completing the surveys than those with dementia. Additionally, improvements in usability ratings may partially reflect survivor bias among participants who remained engaged with the platform over the 52-week period. Furthermore, the SUS and most of the feedback surveys imply the NeuLogiq Platform and the Dreem sleep headband and app as one system encompassing the user's global experience, therefore a low score cannot be attributed to one feature or another. Furthermore, terminology used in questions related to “support” may have been unclear and interpreted differently by participants. Participants received support in the study in various ways (i.e., from a study partner or the site staff). Given that the ePRO surveys were conducted in unmonitored, remote settings, it is likely that misinterpretation occurred. Future studies involving feedback on the NeuLogiq Platform should provide clarity around the system components and the term “support” to truly understand the usability of digital technology at home.

Lastly, caution must be applied when generalizing the results of the current study to other populations. The current study recruited patients from a selection of public and private health clinics across England and Scotland. Populations from other parts of the world might experience different challenges than these when engaging with new technology in a protocol like that reported here. For example, participation might be more challenging in countries with poorer telecommunications infrastructure, upon which the platform currently relies. Notably, a small number (*N* = 2) of participants in the present study were identified as excluded due to unreliable wireless internet connectivity during screening, suggesting that internet access within the recruited population was generally sufficient to support remote longitudinal data collection. If poor wireless connectivity is an issue in future studies that prevent enrolment, the dedicated tablets provided by Cumulus for home-based assessment are SIM card and mobile hotspot compatible (while not necessary for this current study). In addition, the nature of recruitment in the current study may have had a positive bias – in that only those judged likely to be interested and able to participate in this observational study by the recruiting clinician were approached. Interventional clinical trials will likely have broader interest from patients who are motivated by access to potential treatments, and subtle differences in the prospective cohort might imply a different level of feasibility. Furthermore, as biomarker confirmation of Alzheimer's disease was encouraged and not mandatory, some diagnostic heterogeneity within the dementia cohort may have been present, reflecting the pragmatic, real-world design of the study.

## 5 Conclusion

This paper serves as a clear demonstration of the feasibility of collecting digital biomarkers (including cognition, sleep and EEG) in the real-world home setting, over a 52-week study in older adults and patients living with AD dementia. Positive signs of usability (through both subjective ratings and evidence of long-term protocol adherence) indicate the Cumulus NeuLogiq platform is suitable for the objective and frequent evaluation of mild Alzheimer's dementia, in a manner that is practical for patients. Despite encountering minor technical challenges during the initial setup at home, participants exhibited high rates of adherence to the protocol, highlighting the platform's potential for successful integration into their daily lives.

## Data Availability

The original contributions presented in the study are included in the article/supplementary material, further inquiries can be directed to the corresponding author/s. The datasets presented in this article are not readily available because the data collected using the NeuLogiq platform is commercially sensitive and contains proprietary information. Requests to access supporting data will be considered from bona fide researchers upon reasonable request.
