# Supplementary material for: A real-world feasibility study: at home longitudinal use of the Cumulus NeuLogiq® platform for electrophysiological and neurocognitive measures in patients with mild Alzheimer's Disease dementia
Source: Front Digit Health. 2026 Jun 26;8:1840966. doi: 10.3389/fdgth.2026.1840966 (PMC13353088; doi:10.3389/fdgth.2026.1840966)
Supplement: Supplementary file 1 [file Table1.docx]

**Supplementary Tables**

**Supplementary Table 1. Baseline demographics and blood biomarker aggregates per group.**

| **Variable_name** | **Descriptive** | **Controls** | **Dementia** |
| --- | --- | --- | --- |
| Demographics |  |  |  |
| Sex | males:females, males% - females% | 29:31, 48.33% - 51.67% | 37:22, 62.71% - 37.29% |
| Education | Left formal education before age 16 | 4 (6.67%) | 11 (18.64%) |
| Education | Left formal education at age 16 | 7 (11.67%) | 8 (13.56%) |
| Education | Left formal education at age 17/18 | 14 (23.33%) | 11 (18.64%) |
| Education | To undergraduate degree or equivalent level | 25 (41.67%) | 14 (23.73%) |
| Education | To Master's degree or equivalent level | 6 (10.00%) | 9 (15.25%) |
| Education | To PhD or equivalent level | 4 (6.67%) | 6 (10.17%) |
| Age | mean (95%CI), n | 71.15 (69.34, 72.96), n=60 | 73.78 (72.06, 75.50), n=59 |
| Blood biomarkers |  |  |  |
| pTau-217 (pg/mL) | mean (95%CI), n | 0.51 (0.41, 0.61), n=47 | 0.88 (0.71, 1.06), n=47 |
| GFAP (pg/mL) | mean (95%CI), n | 118.97 (100.88, 137.06), n=47 | 161.82 (139.63, 184.02), n=47 |
| NfL (pg/mL) | mean (95%CI), n | 19.60 (15.21, 24.00, n=47 | 24.59 (21.68, 27.51), n=47 |
| Aβ 42/40 | mean (95%CI), n | 0.06 (0.06, 0.07), n=47 | 0.06 (0.06, 0.06), n=47 |
| Assessment |  |  |  |
| ADASCOG | mean (95%CI), n | 8.88 (7.72, 10.05), n=60 | 25.07 (23.03, 27.11), n=59 |
| NART | mean (95%CI), n | 41.40 (40.20, 42.60), n=60 | 36.20 (33.90, 38.50), n=59 |
| ACE III score | mean (95%CI), n | 94.85 (94.07, 95.63), n=60 | 77.59 (75.78, 79.40), n=59 |
| Verbal paired associates | mean (95%CI), n | 26.35 (24.71, 27.99), n=60 | 12.20 (10.67, 13.74), n=59 |
| Coding | mean (95%CI), n | 56.72 (53.06, 60.37), n=60 | 37.78 (34.03, 41.52), n=59 |
| Apathy motivation index | mean (95%CI), n | 1.24 (1.15, 1.33), n=58 | 1.55 (1.45, 1.65), n=55 |
| Geriatric depression score | mean (95%CI), n | 1.09 (0.66, 1.52), n=58 | 3.50 (2.61, 4.39), n=56 |
| Converted total score (SCI) | mean (95%CI), n | 7.45 (6.91, 7.98), n=58 | 7.34 (6.69, 7.99), n=55 |
| Depression scale (DASS) | mean (95%CI), n | 3.41 (2.30, 4.53), n=58 | 6.89 (5.16, 8.62), n=54 |
| Anxiety scale (DASS) | mean (95%CI), n | 3.17 (2.11, 4.23), n=58 | 5.30 (3.78, 6.81), n=54 |
| Stress scale (DASS) | mean (95%CI), n | 6.31 (4.89, 7.73), n=58 | 9.59 (7.59, 11.60), n=54 |
| Cantril's ladder - past | mean (95%CI), n | 7.74 (7.26, 8.22), n=58 | 7.94 (7.43, 8.46), n=54 |
| Cantril's ladder - present | mean (95%CI), n | 7.97 (7.67, 8.26), n=58 | 7.09 (6.53, 7.65), n=54 |
| Cantril's ladder - future | mean (95%CI), n | 7.50 (7.03, 7.97), n=58 | 5.98 (5.27, 6.70), n=54 |

**Supplementary Table 2. Observed means adherence by participant group by stage, expressed as a percentage of total sessions requested in the protocol.**

| **Stage** | **Metric** | **Alzheimer's disease** | **Controls** |
| --- | --- | --- | --- |
| Stage 1 Familiarisation (2 weeks) | N participants | 56 | 57 |
|  | Mean % of sessions opened per participant (95% CI) | 78.59 (71.79, 85.38) | 87.49 (81.50, 93.48) |
|  | Mean number of sessions opened per participant (95% CI) | 6.82 (6.14, 7.50) | 7.77 (7.20, 8.34) |
| Stage 2  Burst (2 weeks) | N participants | 51 | 57 |
|  | Mean % of sessions opened per participant (95% CI) | 69.99 (61.64, 78.33) | 82.11 (75.85, 88.36) |
|  | Mean number of sessions opened per participant (95% CI) | 10.10 (8.79, 11.40) | 12.04 (11.06, 13.01) |
| Stage 3  Fortnightly cycles (month 2-6) | N participants | 51 | 56 |
|  | Mean % of sessions opened per participant (95% CI) | 80.36 (72.77, 87.95) | 91.89 (87.62, 96.16) |
|  | Mean number of sessions opened per participant (95% CI) | 25.06 (22.43, 27.69) | 29.48 (27.87, 31.09) |
| Stage 4  Monthly cycles (month 7-12) | N participants | 45 | 56 |
|  | Mean % of sessions opened per participant (95% CI) | 78.53 (69.80, 87.26) | 92.16 (87.85, 96.47) |
|  | Mean number of sessions opened per participant (95% CI) | 13.91 (12.20, 15.62) | 17.23 (16.22, 18.24) |

**Supplementary Table 3. Observed means adherence by participant group by stage and by session type, expressed as a percentage of total sessions requested in the protocol.**

| **Stage** | **Session type** | **Dementia** | **Controls** | |
| --- | --- | --- | --- | --- |
| Stage 1 | A | 85.71 (78.88, 92.55), n=56 | 90.64 (84.80, 96.48), n=57 | |
|  | B | 82.42 (74.68, 90.17), n=55 | 87.72 (81.25, 94.19), n=57 | |
|  | C | 70.37 (60.92, 79.82), n=54 | 87.27 (81.16, 93.38), n=55 | |
| Stage 2 | A | 77.25 (68.27, 86.24), n=51 | 86.32 (79.80, 92.84), n=57 | |
|  | B | 69.41 (60.34, 78.49), n=51 | 82.11 (75.55, 88.66), n=57 | |
|  | C | 73.14 (63.17, 83.12), n=44 | 82.47 (76.26, 88.68), n=54 | |
| Stage 3 | A | 85.02 (77.63, 92.41), n=51 | 93.67 (89.36, 97.97), n=56 | |
|  | B | 81.66 (73.37, 89.94), n=51 | 91.72 (87.20, 96.24), n=56 |  |
|  | C | 73.80 (63.99, 83.60), n=46 | 91.03 (86.52, 95.55), n=54 |  |
| Stage 4 | A | 83.17 (74.67, 91.68), n=45 | 94.64 (90.21, 99.07), n=56 | |
|  | B | 78.41 (68.89, 87.94), n=45 | 93.11 (88.17, 98.06), n=56 |  |
|  | C | 67.69 (55.14, 80.23), n=38 | 90.18 (86.16, 94.20), n=49 |  |

**Supplementary Table 4. Type III Sum of Squares ANOVA results capturing main and interaction effects on adherence of Stages 2-4.**

| **Variable** | **Sum squares** | **Mean squares** | **Num df** | **Den df** | **F value** | **p value** |
| --- | --- | --- | --- | --- | --- | --- |
| **Diagnosis** | **4064.64** | **4064.64** | **1** | **99.79** | **11.77** | **0.0009** |
| **Stage nr** | **8848.2** | **4424.1** | **2** | **803.33** | **12.81** | **3.3E-06** |
| **Session Type** | **13576.11** | **6788.06** | **2** | **802.29** | **19.65** | **4.7E-9** |
| **Diagnosis:Stage nr** | **3789.85** | **1894.92** | **2** | **803.39** | **5.49** | **0.0043** |
| **Diagnosis:Session Type** | **2421.34** | **1210.67** | **2** | **802.3** | **3.5** | **0.0305** |
| Stage nr:Session Type | 2027.69 | 506.92 | 4 | 800.88 | 1.47 | 0.2101 |

**Note:** *Significant effects are presented in bold.*

**Supplementary Table 5. Mixed effects model’s coefficients.**

| **Fixed effect** | **Estimate** | **Std Error** | **df** | **t value** | **p value** |
| --- | --- | --- | --- | --- | --- |
| (Intercept) | 85.67 | 3.5 | 219.61 | 24.45 | 1.3E-64 |
| Dementia | -7.69 | 4.8 | 175.55 | -1.6 | 0.1112 |
| Stage nr 3: 3 | 7.03 | 2.91 | 802.01 | 2.42 | 0.0158 |
| Stage nr 4: 4 | 8.49 | 2.93 | 802.07 | 2.89 | 0.0039 |
| Session Type B: B | -4.63 | 2.89 | 800.12 | -1.6 | 0.1099 |
| Session Type C: C | -3.05 | 2.96 | 801.93 | -1.03 | 0.3022 |
| Dementia:Stage nr 3 | -0.08 | 2.98 | 801.59 | -0.03 | 0.9788 |
| Dementia:Stage nr 4 | -8.93 | 3.07 | 805.17 | -2.91 | 0.0038 |
| Dementia:Session Type B | -2.75 | 2.97 | 800.12 | -0.93 | 0.3538 |
| Dementia:Session Type C | -8.04 | 3.07 | 803.46 | -2.62 | 0.009 |
| Stage nr 3:Session Type B | 3.32 | 3.59 | 800.12 | 0.93 | 0.3552 |
| Stage nr 4:Session Type B | 2.88 | 3.64 | 800.12 | 0.79 | 0.4286 |
| Stage nr 3:Session Type C | -1.78 | 3.67 | 801.13 | -0.49 | 0.6273 |
| Stage nr 4:Session Type C | -5.84 | 3.77 | 801.62 | -1.55 | 0.1218 |

***Note:*** *The intercept refers to the adherence of controls, in session A at Stage 2.*

**Supplementary Table 6. Estimated marginal means (EMM) of levels per factor for mixed effect model of adherence.**

| **Effect** | **Level factor 1** | **Level factor 2** | **EMM** | **Std Error EMM** | **df** | **lower CI** | **upper CI** |
| --- | --- | --- | --- | --- | --- | --- | --- |
| Diagnosis | Dementia |  | 73.83 | 3.03 | 100.37 | 66.94 | 80.73 |
|  | Controls |  | 88.12 | 2.86 | 99.16 | 81.62 | 94.63 |
| Stage nr | Stage 2 |  | 77.46 | 2.25 | 135.66 | 72.01 | 82.92 |
|  | Stage 3 |  | 84.96 | 2.25 | 135.73 | 79.51 | 90.42 |
|  | Stage 4 |  | 80.5 | 2.28 | 143.58 | 74.97 | 86.04 |
| Session Type | Session A |  | 85.49 | 2.25 | 135.28 | 80.05 | 90.94 |
|  | Session B |  | 81.56 | 2.25 | 135.28 | 76.11 | 87.01 |
|  | Session C |  | 75.88 | 2.29 | 144.13 | 70.34 | 81.42 |
| Diagnosis:Stage nr | Dementia | Stage 2 | 71.82 | 3.27 | 136.61 | 63.06 | 80.58 |
|  | Dementia | Stage 3 | 79.28 | 3.27 | 135.63 | 70.53 | 88.03 |
|  | Dementia | Stage 4 | 70.4 | 3.35 | 148.44 | 61.44 | 79.35 |
|  | Controls | Stage 2 | 83.11 | 3.08 | 134.53 | 74.85 | 91.37 |
|  | Controls | Stage 3 | 90.65 | 3.09 | 135.82 | 82.37 | 98.93 |
|  | Controls | Stage 4 | 90.61 | 3.11 | 137.99 | 82.3 | 98.93 |
| Diagnosis:Session Type | Dementia | Session A | 80.15 | 3.27 | 136.13 | 71.39 | 88.9 |
|  | Dementia | Session B | 74.84 | 3.27 | 136.13 | 66.08 | 83.59 |
|  | Dementia | Session C | 66.51 | 3.34 | 147.84 | 57.57 | 75.45 |
|  | Controls | Session A | 90.84 | 3.08 | 134.32 | 82.58 | 99.1 |
|  | Controls | Session B | 88.28 | 3.08 | 134.32 | 80.02 | 96.54 |
|  | Controls | Session C | 85.25 | 3.12 | 139.83 | 76.91 | 93.59 |

**Supplementary Table 7. Post-hoc contrasts of linear mixed model fitted to adherence.**

| **Contrast** | **Estimate** | **Std Error** | **df** | **t ratio** | **p value** | **Corrected p** |
| --- | --- | --- | --- | --- | --- | --- |
| **Controls – Dementia** | **14.29** | **4.17** | **99.79** | **3.43** | **0.0009** | **0.0029** |
| **2 – 3** | **-7.5** | **1.49** | **801.52** | **-5.03** | **6E-07** | **3.9E-06** |
| 2 – 4 | -3.04 | 1.54 | 805.07 | -1.97 | 0.0486 | 0.0632 |
| **3 – 4** | **4.46** | **1.53** | **803.49** | **2.91** | **0.0038** | **0.0074** |
| **A – B** | **3.94** | **1.48** | **800.12** | **2.65** | **0.0081** | **0.013** |
| **A - C** | **9.61** | **1.54** | **803.46** | **6.26** | **6.4E-10** | **8.3E-09** |
| **B – C** | **5.68** | **1.54** | **803.46** | **3.69** | **0.0002** | **0.0009** |
| (Controls - Dementia) - (2 – 3) | -0.08 | 2.98 | 801.59 | -0.03 | 0.9788 | 0.9788 |
| **(Controls - Dementia) - (2 – 4)** | **-8.93** | **3.07** | **805.17** | **-2.91** | **0.0038** | **0.0074** |
| **(Controls - Dementia) - (3 – 4)** | **-8.85** | **3.06** | **803.5** | **-2.89** | **0.004** | **0.0074** |
| (Controls - Dementia) - (A – B) | -2.75 | 2.97 | 800.12 | -0.93 | 0.3538 | 0.3833 |
| **(Controls - Dementia) - (A – C)** | **-8.04** | **3.07** | **803.46** | **-2.62** | **0.009** | **0.013** |
| (Controls - Dementia) - (B – C) | -5.29 | 3.07 | 803.46 | -1.72 | 0.0852 | 0.1007 |

**Note:** *Contrasts that survived correction for multiple comparisons are presented in bold.*

**Supplementary Table 8: Baseline demographics and blood biomarker aggregates per group, split by withdrawals.**

| **Variables** | **Controls** |  | **Dementia** |  |
| --- | --- | --- | --- | --- |
| **Demographics** | **Completed** | **Withdrawn** | **Completed** | **Withdrawn** |
| N | 54 | 6 | 43 | 16 |
| Sex – males:females, males% - females% | 27:27, 50.00% - 50.00% | 2:4, 33.33% - 66.67% | 28:15, 65.12% - 34.88% | 9:7, 56.25% - 43.75% |
| Education – Left formal education before age 16 n (%) | 3 (5.56%) | 1 (16.67%) | 9 (20.93%) | 2 (12.50%) |
| Education – Left formal education at age 16, n (%) | 6 (11.11%) | 1 (16.67%) | 6 (13.95%) | 2 (12.50%) |
| Education – Left formal education at age 17/18, n (%) | 13 (24.07%) | 1 (16.67%) | 10 (23.26%) | 1 (6.25%) |
| Education – To undergraduate degree or equivalent level, n(%) | 22 (40.74%) | 3 (50.00%) | 10 (23.26%) | 4 (25.00%) |
| Education – To Master's degree or equivalent level, n(%) | 6 (11.11%) | 0 (0.00%) | 4 (9.30%) | 5 (31.25%) |
| Education – To PhD or equivalent level, n(%) | 4 (7.41%) | 0 (0.00%) | 4 (9.30%) | 2 (12.50%) |
| Age | 70.69 (68.79, 72.58), n=54 | 75.33 (70.21, 80.46), n=6 | 72.72 (70.82, 74.62), n=43 | 76.62 (73.17, 80.08), n=16 |
| Blood biomarkers |  |  |  |  |
| pTau-217 (pg/mL) | 0.52 (0.41, 0.62), n=46 | 0.29, n=1 | 0.93 (0.74, 1.12), n=41 | 0.56 (0.24, 0.88), n=6 |
| GFAP (pg/mL) | 120.02 (101.66, 138.39), n=46 | 70.39, n=1 | 165.75 (140.78, 190.71), n=41 | 135.03 (106.72, 163.34), n=6 |
| NfL (pg/mL) | 19.79 (15.32, 24.26), n=46 | 10.72, n=1 | 25.11 (21.94, 28.28), n=41 | 21.05 (13.80, 28.31), n=6 |
| Aβ 42/40 | 0.06 (0.06, 0.07), n=46 | 0.07, n=1 | 0.06 (0.06, 0.06), n=41 | 0.06 (0.06, 0.07), n=6 |
| Assessment |  |  |  |  |
| ADASCOG | 8.41 (7.25, 9.56), n=54 | 13.17 (8.95, 17.38), n=6 | 23.98 (21.61, 26.35), n=43 | 28.00 (24.26, 31.74), n=16 |
| NART | 41.69 (40.46, 42.91), n=54 | 38.83 (34.21, 43.46), n=6 | 35.37 (32.46, 38.29), n=43 | 38.44 (35.30, 41.58), n=16 |
| ACE III score | 95.07 (94.23, 95.92), n=54 | 92.83 (91.66, 94.01), n=6 | 78.58 (76.75, 80.41), n=43 | 74.94 (70.58, 79.30), n=16 |
| Verbal paired associates | 26.87 (25.18, 28.56), n=54 | 21.67 (16.59, 26.74), n=6 | 13.23 (11.47, 14.99), n=43 | 9.44 (6.68, 12.19), n=16 |
| Coding | 56.98 (53.02, 60.94), n=54 | 54.33 (45.78, 62.89), n=6 | 36.58 (32.35, 40.82), n=43 | 41.00 (33.17, 48.83), n=16 |
| Apathy motivation index | 1.23 (1.14, 1.33), n=54 | 1.35 (1.14, 1.56), n=4 | 1.57 (1.44, 1.69), n=43 | 1.49 (1.32, 1.65), n=12 |
| Geriatric depression score | 1.11 (0.65, 1.57), n=54 | 0.75 (-0.19, 1.69), n=4 | 3.42 (2.37, 4.46), n=43 | 3.77 (2.01, 5.53), n=13 |
| Converted total score (SCI) | 7.44 (6.89, 7.98), n=54 | 7.58 (4.72, 10.43), n=4 | 7.43 (6.74, 8.13), n=43 | 7.01 (5.30, 8.71), n=12 |
| Depression scale (DASS) | 3.22 (2.06, 4.39), n=54 | 6.00 (2.80, 9.20), n=4 | 6.33 (4.44, 8.22), n=42 | 8.83 (4.73, 12.94), n=12 |
| Anxiety scale (DASS) | 3.07 (1.97, 4.18), n=54 | 4.50 (0.46, 8.54), n=4 | 5.57 (3.81, 7.34), n=42 | 4.33 (1.37, 7.30), n=12 |
| Stress scale (DASS) | 6.22 (4.78, 7.67), n=54 | 7.50 (-0.07, 15.07), n=4 | 9.52 (7.18, 11.86), n=42 | 9.83 (5.87, 13.80), n=12 |
| Cantril's ladder - past | 7.70 (7.19, 8.22), n=54 | 8.25 (7.31, 9.19), n=4 | 7.90 (7.29, 8.52), n=42 | 8.08 (7.20, 8.97), n=12 |
| Cantril's ladder - present | 7.96 (7.65, 8.27), n=54 | 8.00 (7.20, 8.80), n=4 | 7.19 (6.54, 7.85), n=42 | 6.75 (5.69, 7.81), n=12 |
| Cantril's ladder - future | 7.50 (7.00, 8.00), n=54 | 7.50 (6.23, 8.77), n=4 | 6.10 (5.31, 6.89), n=42 | 5.58 (3.89, 7.28), n=12 |

**Note:** *Means, 95%CI and the sample size are reported for all variables except for Sex and Age where percentages are reported.*

**Supplementary Table 9: Number of responses per rating in the surveys ‘Background on technology usage’ and ‘Technology Feedback’ at Baseline, Weeks 2, 26 and 52.**

| **Question** | **Ratings** | | | | | **Timepoint** |
| --- | --- | --- | --- | --- | --- | --- |
| *Background on technology usage* |  | | | | |  |
| **How often does the participant use technology such as smartphones, tablets and computers?** | **1 (Rarely/never)** | **2** | **3** | **4** | **5 (Every day)** | Baseline |
| Control (N) | 1 | 4 | 1 | 4 | 48 |  |
| Dementia (N) | 3 | 3 | 10 | 5 | 36 |  |
| **How confident do you feel about using technology such as smartphones, tablets and computers?** | **1 (Not at all confident)** | **2** | **3** | **4** | **5 (Very confident)** | Baseline |
| Control (N) | 1 | 2 | 19 | 14 | 23 |  |
| Dementia (N) | 4 | 13 | 19 | 14 | 7 |  |
| **How much help do you feel you would need to get started with a new technology that is like a smartphone, tablet or computer?** | **1 (A lot of help)** | **2** | **3** | **4** | **5 (No help at all)** | Baseline |
| Control (N) | 2 | 8 | 20 | 16 | 13 |  |
| Dementia (N) | 16 | 12 | 17 | 9 | 3 |  |
| *Technology Feedback at Baseline* |  |  |  |  |  |  |
| **How difficult/easy was it to learn how to use the platform during the training session?** | **1 (Very difficult)** | **2** | **3** | **4** | **5 (Very easy)** | Baseline |
| Control (N) | 0 | 6 | 15 | 22 | 15 |  |
| Dementia (N) | 5 | 9 | 25 | 15 | 3 |  |
| **How confident do you feel about using this technology at home?** | **1 (Not at all confident)** | **2** | **3** | **4** | **5 (Very confident)** | Baseline |
| Control (N) | 4 | 5 | 11 | 29 | 9 |  |
| Dementia (N) | 5 | 10 | 24 | 16 | 2 |  |
|  |  |  |  |  |  |  |
| *Technology Feedback* |  |  |  |  |  |  |
| **How much support was needed to enable you to complete each session?** | **1 (A lot of support)** | **2** | **3** | **4** | **5 (No/little support)** |  |
| Control (N) | 1 | 7 | 9 | 7 | 29 | Week 2 |
| Dementia (N) | 8 | 15 | 9 | 4 | 4 | Week 2 |
| Control (N) | 1 | 1 | 3 | 11 | 38 | Week 26 |
| Dementia (N) | 6 | 7 | 9 | 5 | 11 | Week 26 |
| Control (N) | 0 | 1 | 2 | 8 | 37 | Week 52 |
| Dementia (N) | 6 | 9 | 4 | 9 | 6 | Week 52 |
| **Thinking about the headset and tablet together, how would you rate ease of use?** | **1 (Very difficult)** | **2** | **3** | **4** | **5 (Very easy)** |  |
| Control (N) | 3 | 8 | 14 | 19 | 9 | Week 2 |
| Dementia (N) | 1 | 9 | 21 | 7 | 1 | Week 2 |
| Control (N) | 2 | 4 | 9 | 29 | 10 | Week 26 |
| Dementia (N) | 1 | 5 | 17 | 9 | 6 | Week 26 |
| Control (N) | 2 | 2 | 8 | 25 | 11 | Week 52 |
| Dementia (N) | 0 | 7 | 10 | 15 | 2 | Week 52 |
| **How do you find each session in terms of fatigue?** | **1 (Very tiring)** | **2** | **3** | **4** | **5 (Not tiring at all)** |  |
| Control (N) | 0 | 4 | 12 | 13 | 24 | Week 2 |
| Dementia (N) | 2 | 5 | 11 | 12 | 9 | Week 2 |
| Control (N) | 3 | 3 | 5 | 13 | 30 | Week 26 |
| Dementia (N) | 1 | 4 | 12 | 8 | 13 | Week 26 |
| Control (N) | 1 | 3 | 4 | 14 | 26 | Week 52 |
| Dementia (N) | 1 | 5 | 4 | 8 | 16 | Week 52 |
| **How well does the session fit in to your daily schedule?** | **1 (Not easy to fit in)** | **2** | **3** | **4** | **5 (Easy to fit in)** |  |
| Control (N) | 1 | 6 | 14 | 14 | 18 | Week 2 |
| Dementia (N) | 1 | 1 | 14 | 9 | 15 | Week 2 |
| Control (N) | 4 | 2 | 12 | 9 | 27 | Week 26 |
| Dementia (N) | 0 | 1 | 13 | 11 | 13 | Week 26 |
| Control (N) | 2 | 3 | 3 | 13 | 27 | Week 52 |
| Dementia (N) | 0 | 2 | 6 | 5 | 21 | Week 52 |
| **How is your sleep quality on the nights when you wear the Dreem headband, compared to your usual sleep quality (not wearing the Dreem headband)?** | **1 (Worse than usual)** | **2** | **3** | **4** | **5 (Better than usual)** |  |
| Control (N) | 10 | 17 | 23 | 2 | 0 | Week 2 |
| Dementia (N) | 3 | 10 | 21 | 1 | 3 | Week 2 |
| Control (N) | 17 | 15 | 19 | 1 | 0 | Week 26 |
| Dementia (N) | 2 | 10 | 22 | 1 | 0 | Week 26 |
| Control (N) | 19 | 13 | 14 | 0 | 1 | Week 52 |
| Dementia (N) | 3 | 5 | 22 | 1 | 1 | Week 52 |

**Supplementary Table 10. Statistical results from the Technology Feedback survey at Baseline, Weeks 2, 26 and 52.**

| **Variable name** | **Survey Type and Timepoint** | **Dementia**  [mean (95%CI), n] | **Controls**  [mean (95%CI), n] | **t statistic** | **p-value** | **Corrected**  **p-value** | **df** | **Cohens d** |
| --- | --- | --- | --- | --- | --- | --- | --- | --- |
| Usage frequency of technology | Technology Feedback at Baseline | 4.19 (3.88, 4.51), n=57 | 4.62 (4.38, 4.87), n=58 | -2.1 | 0.038 | 0.038 | 113 | -0.39 |
| **Confidence in using technology** | **Technology Feedback at Baseline** | **3.12 (2.83, 3.41), n=57** | **3.95 (3.69, 4.21), n=59** | **-4.18** | **5.68x10^-05^** | **8.52x10^-05^** | **114** | **-0.78** |
| **Help needed to get started with a new technology** | **Technology Feedback at Baseline** | **2.49 (2.18, 2.81), n=57** | **3.51 (3.23, 3.79), n=59** | **-4.76** | **5.75x10^-06^** | **1.73x10^-05^** | **114** | **-0.88** |
| **Difficulty learning to use the technology** | **Feedback at Baseline** | **2.04 (1.78, 2.29), n=57** | **2.79 (2.55, 3.04), n=58** | **-4.17** | **6.04x10 ^-05^** | **1.2x10^-04^** | **113** | **-0.78** |
| **Confidence in using the technology** | **Feedback at Baseline** | **2.00 (1.75, 2.25), n=57** | **2.59 (2.31, 2.86), n=58** | **-3.05** | **0.003** | **0.003** | **113** | **-0.57** |
| Ease of use of headset and tablet | Technology Feedback at Week 2 | 1.95 (1.71, 2.19), n=40 | 2.43 (2.13, 2.74), n=53 | -2.34 | 0.022 | 0.055 | 91 | -0.5 |
| Ease of use of headset and tablet | Technology Feedback at Week 26 | 2.37 (2.05, 2.69), n=38 | 2.76 (2.50, 3.02), n=54 | -1.88 | 0.063 | 0.1294 | 90 | -0.4 |
| Ease of use of headset and tablet | Technology Feedback at Week 52 | 2.35 (2.06, 2.65), n=34 | 2.85 (2.58, 3.13), n=48 | -2.39 | 0.019 | 0.055 | 80 | -0.54 |
| Fatigue during session | Technology Feedback at Week 2 | 2.55 (2.20, 2.90), n=40 | 3.08 (2.81, 3.34), n=53 | -2.37 | 0.02 | 0.055 | 91 | -0.49 |
| Fatigue during session | Technology Feedback at Week 26 | 2.74 (2.38, 3.10), n=38 | 3.19 (2.87, 3.50), n=54 | -1.84 | 0.069 | 0.1294 | 90 | -0.39 |
| Fatigue during session | Technology Feedback at Week 52 | 2.97 (2.56, 3.38), n=34 | 3.27 (2.99, 3.56), n=48 | -1.22 | 0.226 | 0.3767 | 80 | -0.27 |
| Session fit in daily schedule | Technology Feedback at Week 2 | 2.90 (2.58, 3.22), n=40 | 2.79 (2.50, 3.09), n=53 | 0.48 | 0.633 | 0.7304 | 91 | 0.1 |
| Session fit in daily schedule | Technology Feedback at Week 26 | 2.95 (2.66, 3.23), n=38 | 2.98 (2.65, 3.32), n=54 | -0.14 | 0.886 | 0.886 | 90 | -0.03 |
| Session fit in daily schedule | Technology Feedback at Week 52 | 3.32 (3.00, 3.65), n=34 | 3.25 (2.94, 3.56), n=48 | --0.31 | 0.756 | 0.81 | 80 | 0.07 |
| Sleep quality when wearing Dreem sleep headband compared to usual sleep quality | Technology Feedback at Week 2 | -3.33 (-10.29, 3.64), n=40 | -0.58 (-4.34, 3.17), n=53 | -0.72 | 0.472 | 0.5937 | 91 | -0.15 |
| Sleep quality when wearing Dreem sleep headband compared to usual sleep quality | Technology Feedback at Week 26 | -6.39 (-15.23, 2.44), n=38 | -2.67 (-7.81, 2.48), n=54 | -0.76 | 0.449 | 0.5937 | 90 | -0.16 |
| Sleep quality when wearing Dreem sleep headband compared to usual sleep quality | Technology Feedback at Week 52 | -4.24 (-12.41, 3.94), n=34 | -1.15 (-5.28, 2.99), n=48 | -0.72 | 0.475 | 0.5937 | 80 | -0.15 |
| **Support needed to enable session completion** | Technology Feedback at Week 2 | **1.52 (1.15, 1.90), n=40** | **3.06 (2.73, 3.38), n=53** | **-6.05** | **3.13x10^-08^** | **2.35x10^-07^** | **91** | **-1.27** |
| **Support needed to enable session completion** | Technology Feedback at Week 26 | **2.21 (1.75, 2.67), n=38** | **3.56 (3.33, 3.78), n=54** | **-5.6** | **2.27x10^-07^** | **1.13x10^-06^** | **90** | **-1.13** |
| **Support needed to enable session completion** | Technology Feedback at Week 52 | **2.00 (1.52, 2.48), n=34** | **3.69 (3.50, 3.87), n=48** | **-7.25** | **2.36x10^-10^** | **3.54x10^-09^** | **80** | **-1.53** |

**Note:** *Significant differences are presented in bold.*

**Supplementary figures**

Supplementary Figure 1: Percentages of responses per group to the ePRO System Usability Scale questionnaire at Week 26.


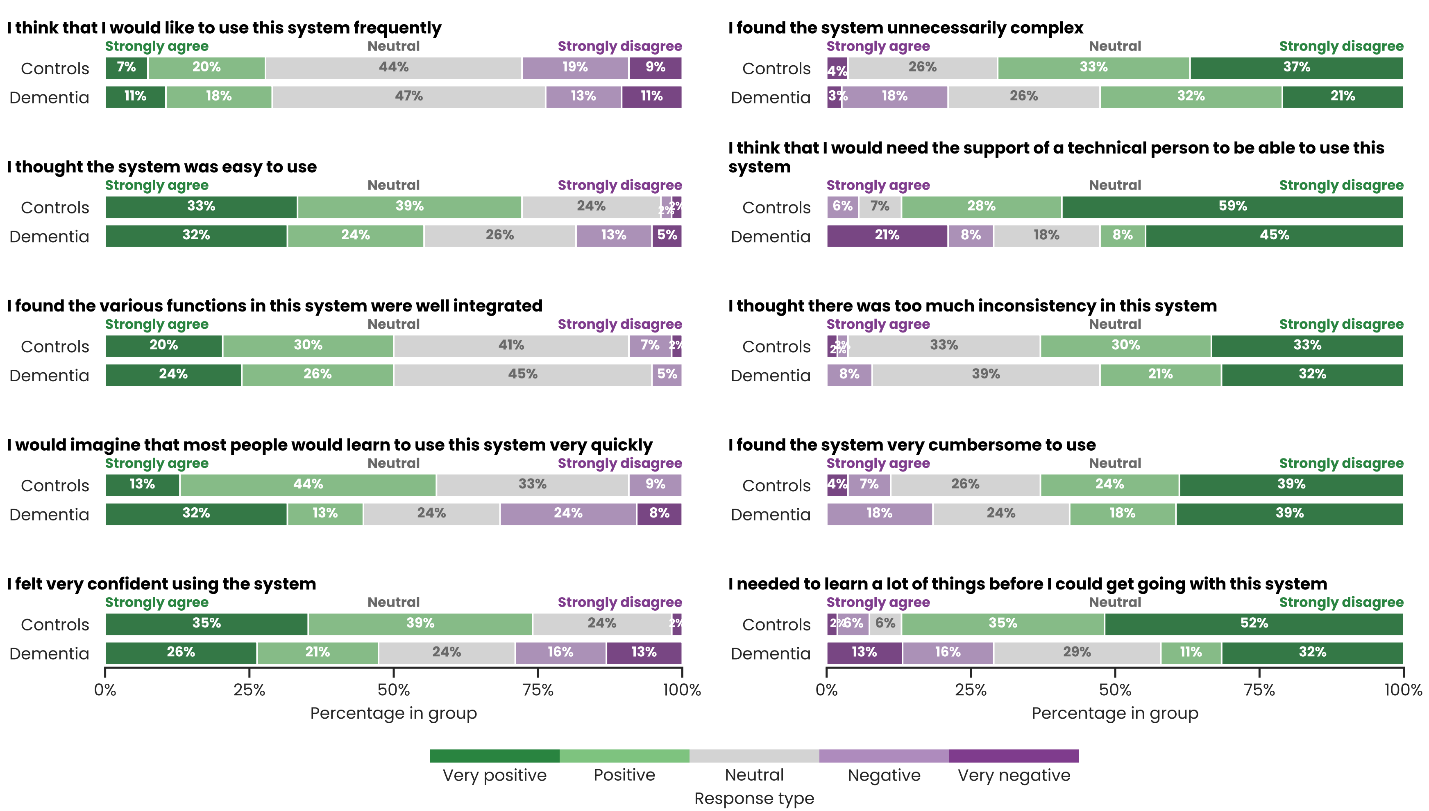


*Note: Percentages per group may not total 100% due to rounding.*

Supplementary Figure 2: Percentages of responses per group to the ePRO System Usability Scale questionnaire at Week 52.


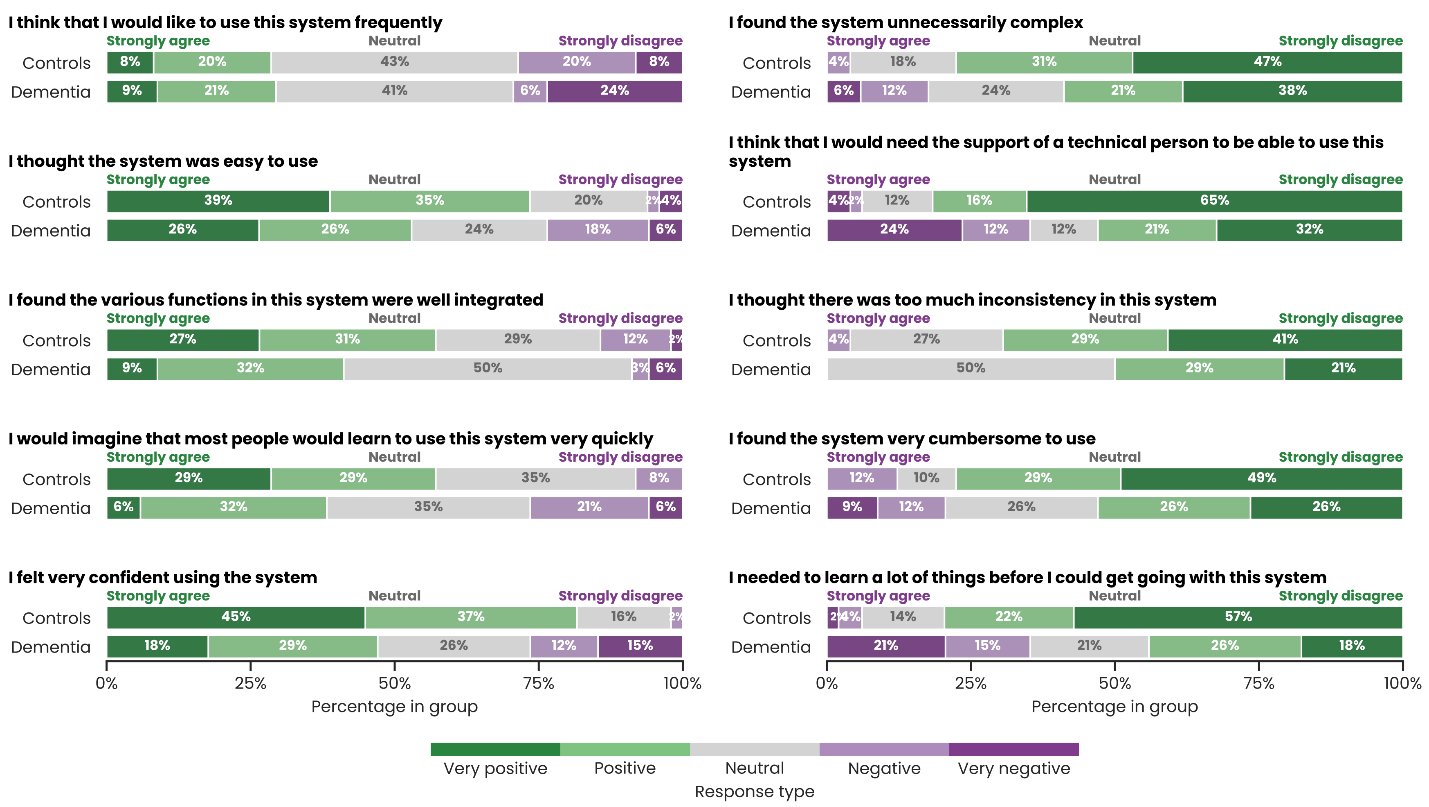


***Note:*** *Percentages per group may not total 100% due to rounding.*
